# Improving Properties of Podophyllic Aldehyde-Derived Cyclolignans: Design, Synthesis and Evaluation of Novel Lignohydroquinones, Dual-Selective Hybrids against Colorectal Cancer Cells

**DOI:** 10.3390/pharmaceutics15030886

**Published:** 2023-03-09

**Authors:** Ángela-Patricia Hernández, Paula Díez, Pablo A. García, Martín Pérez-Andrés, Anzhela Veselinova, Pablo G. Jambrina, Arturo San Feliciano, David Díez, Manuel Fuentes, Mᵃ Ángeles Castro

**Affiliations:** 1 Department of Pharmaceutical Sciences: Organic Chemistry, Faculty of Pharmacy, University of Salamanca, CIETUS, IBSAL, 37007 Salamanca, Spain; 2 Department of Medicine and General Cytometry Service-Nucleus, CIBERONC CB16/12/00400, Cancer Research Centre (IBMCC/CSIC/USAL/IBSAL), 37007 Salamanca, Spain; 3 Departamento de Química Física, Facultad de Ciencias Químicas, Universidad de Salamanca, 37008 Salamanca, Spain; 4 Programa de Pós-Graduaçao em Ciências Farmacêuticas, Universidade do Vale do Itajaí, UNIVALI, Itajaí 88302-202, SC, Brazil; 5 Departamento de Química Orgánica, Facultad de Ciencias Químicas, Universidad de Salamanca, 37008 Salamanca, Spain; 6 Proteomics Unit, Cancer Research Centre (IBMCC/CSIC/USAL/IBSAL), 37007 Salamanca, Spain

**Keywords:** podophyllotoxin, terpenylhydroquinones, hybridization, lignohydroquinones, flow cytometry, cytotoxicity, cell cycle arrest, colchicine binding site

## Abstract

New lignohydroquinone conjugates (L-HQs) were designed and synthesized using the hybridization strategy, and evaluated as cytotoxics against several cancer cell lines. The L-HQs were obtained from the natural product podophyllotoxin and some semisynthetic terpenylnaphthohydroquinones, prepared from natural terpenoids. Both entities of the conjugates were connected through different aliphatic or aromatic linkers. Among the evaluated hybrids, the L-HQ with the aromatic spacer clearly displayed the in vitro dual cytotoxic effect derived from each starting component, retaining the selectivity and showing a high cytotoxicity at short (24 h) and long (72 h) incubation times (4.12 and 0.0450 µM, respectively) against colorectal cancer cells. In addition, the cell cycle blockade observed by flow cytometry studies, molecular dynamics, and tubulin interaction studies demonstrated the interest of this kind of hybrids, which docked adequately into the colchicine binding site of tubulin despite their large size. These results prove the validity of the hybridization strategy and encourage further research on non-lactonic cyclolignans.

## 1. Introduction

Cancer continues to be one of the most important public health problems and searching for new therapies against this complex disease is a current challenge for Medicinal Chemistry [1]. Although there are new perspectives in cancer chemoprevention, developing new treatments is essential to overcome drug resistance or the lack of effectiveness of existing drugs. Within this context, natural products are promising and efficient starting materials in the synthesis and development of new therapies. There are many natural products and semisynthetic derivatives, acting through different mechanisms of action, which are used in different therapeutic contexts such as antiparasitic [2] or cardiovascular diseases [3], or for the treatment of various types of cancer [4,5,6,7]. Attending at the origin of compounds involved in cancer therapy, around 60% of the current anticancer drugs are obtained from natural sources [8,9], including the well-known terrestrial environment and the still partially known sea bottom [10,11]. Natural products also provide a variety of specific molecular mechanisms due to their unique structures, even exerting a selectivity toward the most aggressive types of cancer [12,13]. Although natural products can be potent and biologically selective in vitro [14], their clinical application may lead to undesired problems, such as low bioavailability or toxicity, that need to be solved with formulation improvements or conjugation with other molecules [15,16].

Hybridization (also named conjugation) is a current strategy in rational drug design to generate new multifunctional compounds by connecting diverse structures. It provides a wide number of combinations, which increase greatly the natural variability [17,18]. The combination of different chemical entities with expected synergistic or dual effects also constitutes a promising strategy to avoid resistances or to discover new treatments in cancer therapy [19]. Thus, different natural products with diverse structures, origins, and mechanisms of action have been combined to create new bioactive entities [20,21,22,23,24,25,26]. Among the variety of natural products, lignans have contributed to remarkable advances in the discovery of new plant-derived drugs, particularly naphthalene cyclolignans, whose main representative is podophyllotoxin [27].

**Figure 1:**
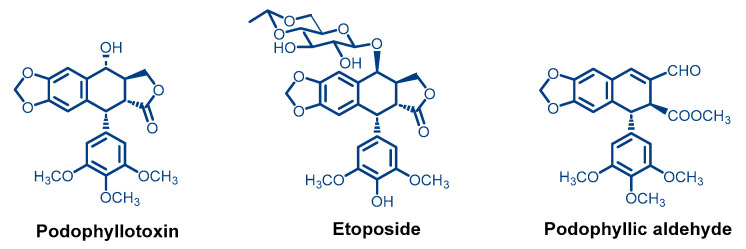
Chemical structures of podophyllotoxin and its derivatives etoposide and podophyllic aldehyde.

Podophyllotoxin, **1** (Figure 1), isolated from the rhizome resin of several *Podophyllum* sp. [28], was commonly used in traditional medicine [29] and became a relevant lead compound in the discovery of new anticancer drugs [30]. The structural changes in the podophyllotoxin skeleton led to clinically used molecules such as etoposide, teniposide, or etopophos [31]. These podophyllotoxin-semisynthetic derivatives could be considered hybrids or conjugates of podophyllotoxin and a glycosidic moiety (Figure 1). Many other cytotoxic natural products of varied biogenetic types (coumarins, isoflavones, bile acids, and many others) have also been combined with podophyllotoxin to look for better antineoplastic derivatives [32]. Our research group has been involved for years in the chemical transformations of podophyllotoxin [33,34,35,36] and our works led to a cytotoxic and a fairly selective derivative that we called podophyllic aldehyde (Figure 1) [37].

The interesting antineoplastic results provided by podophyllic aldehyde, a non-lactonic cyclolignan, made it our lead for further structural modifications [38], including hybridization with other natural products such as terpenylhydroquinone and purine derivatives [38,39]. Now, a new family of hybrids have been designed and synthesized by joining this lead compound with other terpenylhydroquinones. Both scaffolds, cyclolignans and terpenylhydroquinones, show cytotoxic effects but present different mechanisms of action that can be combined in the conjugates to enhance the pharmacological activities of these natural derived compounds. Several biological assays have been carried out to display how hybridization can improve the cytotoxic and selectivity properties of the starting compounds. The background and the approach to the present work are pointed out below.

### Chemistry Approach and Objective

**Figure 2:**
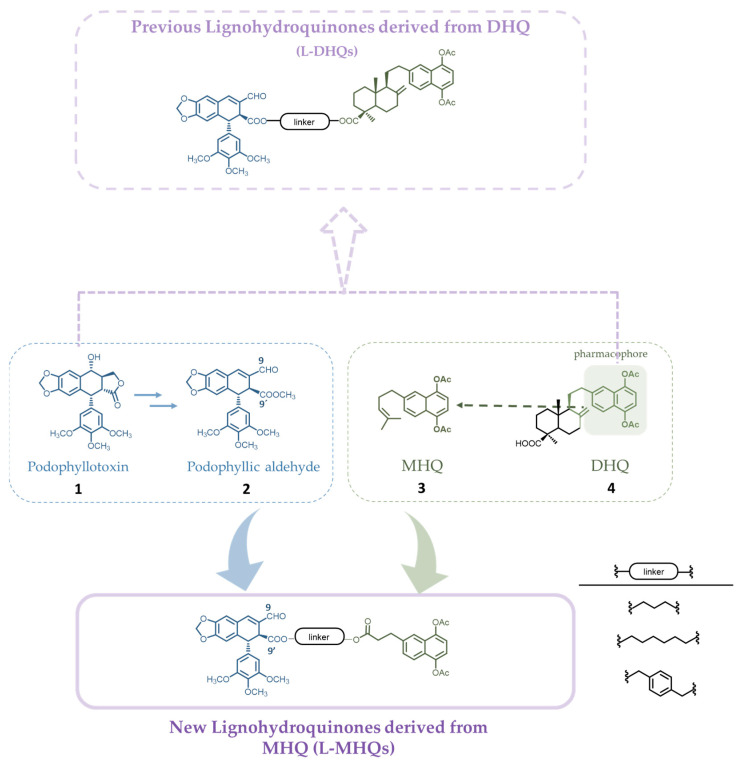
Schematic representation of the design of the new simplified hybrids lignomonoterpenylnaphthohydroquinones (L-MHQs), from cyclolignans (**1** and **2**) and monoterpenylnaphthohydroquinones (MHQ **3**) as a simplified model of diterpenylnaphthohydroquinones (DHQ **4**).

In previous work [38], we prepared hybrids, generically named lignohydroquinones (L-HQs), by linking cyclolignans (L) and a diterpenylnaphthohydroquinone (DHQ, Figure 2) through aliphatic and aromatic linkers. The DHQ was obtained by us from the natural diterpenoid myrceocommunic acid [40] and the cyclolignan scaffold was obtained by transforming podophyllotoxin (**1**) into the podophyllic aldehyde (**2**) (Figure 2) [33].

We found that some of those L-HQs with a diterpenoid rest (named L-DHQs) improved the antineoplastic cytotoxicity of the parent compounds, preserving the tubulin inhibition effect and the apoptosis induction associated with podophyllic aldehyde but with a faster effect, which could be associated with the presence of the terpenylquinone moiety (Figure 2) [38]. The approach of the present work is based on these previous results.

Among the many existing strategies in the search for new drugs, there is the simplification of the original structure of the drug to locate the pharmacophore, defined as the structural elements necessary for the compound to maintain its pharmacological activity. Bearing this in mind, the activity could be optimized by synthesizing new agents whose structures include this pharmacophoric grouping. In the case of our previous L-DHQs [38], it seemed that the acetylated naphthohydroquinonic part of the DHQ would be responsible for the rapid activity acting as a possible pharmacophore (Figure 2).

Thus, we decided to simplify the terpenylquinonic fragment, shortening the distance between the cyclolignan and this hypothetical pharmacophore. Our previous experience, not only with DHQs but also with monoterpenylnaphthohydroquinones (MHQs, Figure 2) and their good cytotoxicity results [41,42], motivated the incorporation of MHQ within the family of L-HQs to obtain the hybrids (named L-MHQs) presented here. Hence, we explored the importance of this scaffold for the bioactivity by attaching a MHQ at the C-9’ position of the cyclolignan, maintaining the α,β-unsaturated carbonyl at the C-9 position (Figure 2). To join both moieties, we chose the same aliphatic and aromatic linkers used in our previous reports to explore how the proximity of the scaffolds (shorter or longer aliphatic linker) or how the conformational restriction (aromatic linker) may influence the final activity and selectivity. These new conjugates, L-MHQs, were tested for their tumoral cytotoxicity to explore how the two moieties included in the final hybrids, L and MHQ, contribute to the progression of cell death. At the same time, the role played by the linkers used was considered too.

## 2. Materials and Methods

### 2.1. Chemical Methods

^1^H and ^13^C nuclear magnetic resonance (NMR) experiments were recorded on Bruker AC 200 (200 or 50.3 MHz, respectively) or Bruker Avance 400DRX (400 or 100 MHz) spectrometers in CDCl_3_ using the residual solvent signal as the reference. Chemical shift (δ) values are expressed in ppm, followed by multiplicity (*s* for singlet, *d* for doublet, *dd* for doublet of doublets, *t* for triplet, *m* for multiplet, and *ABq* for AB quartet) for coupling constants (J) in Hz, the normalized integration value, and assigned atoms in brackets. Only representative chemical shift values of ^1^H NMR data are described and assigned in this section for the new compounds. Complete ^1^H and ^13^C NMR assignments are included in Tables S5–S12 and Figures S2–S13.

Infrared (IR) spectra were obtained on a Nicolet Impact 410 spectrophotometer in NaCl film. High-Resolution Mass Spectrometry (HRMS) was run on a QSTAR XL Q-TOF (Applied Biosystems) using electrospray ionization (ES) at 5500 V with an HPLC Agilent 1100 chromatograph. Solvents and reagents were purified by standard procedures as necessary and the chlorinated solvents, including CDCl_3_, were filtered through NaHCO_3_ prior to its use, in order to eliminate acid traces. DMF was dried over molecular sieves and treated with K_2_CO_3_ for the same reason. Column chromatography (CC) purifications were performed using silica gel 60 (40–63 μm, 230–400 mesh, Merck, Rahway, NJ, USA).

#### 2.1.1. Starting Materials

Podophyllotoxin, **1**, was isolated from the rhizome resin of *Podophyllum emodi* (synonym of *Podophyllum hexandrum* Royle, Berberidaceae) as previously described by us [35]. Podophyllic aldehyde, **2**, and acetonide **5** were obtained from **1** following the procedures described by our group [39]. For MHQ precursors **3** and **6**, procedures described by us were followed [43,44].

#### 2.1.2. General Method for Formation of Esters

A solution of acetonide **5** or monoterpenylhydroquinone (MHQ) **6** and K_2_CO_3_ in dry DMF (1.5 mL) was stirred at room temperature for 15 min. Then, the corresponding dibromo-derivative was added and stirred for 2 h more. The reaction mixture was diluted with EtOAc and water and the organic phase was washed with brine, dried over Na_2_SO_4_, and filtered. The reaction products were purified by CC on silica gel to give the corresponding alkylated acetonides **7**–**9** and MHQs **10**–**12**, respectively. The same reaction conditions were applied to obtain acetonides **13**–**15**, either starting from acetonides **7**–**9** by adding MHQ **6** or starting from MHQs **10**–**12**, in which case acetonide **5** was added.

##### Acetonide **7**

From **5** (200 mg, 0.424 mmol), K_2_CO_3_ (174 mg, 1.26 mmol), 1,3-dibromopropane (136 μL, 1.26 mmol). The reaction product was purified by CC, eluting with 30% EtOAc/CH_2_Cl_2_ to give compound **7** (136 mg, 55%). ^1^H NMR: δ for the acetonide fragment: ^1^H NMR: δ 1.48, (*s*, 3H, H12), 1.28 (*s*, 3H, H13), 3.77 (*s*, 6H, H10′, H12′), 3.82 (*s*, 3H, H11′), 6.29 (*s*, 2H, H2′, H6′), 6.38 (*s*, 1H, H3), 6.92 (s, 1H, H6); for the linker: 2.10 (*m*, 2H, O-CH_2_-CH_2_-CH_2_Br), 3.38 (*t*, 6.8, 2H, O-CH_2_-CH_2_-CH_2_Br), 4.04 (m, 2H, O-CH_2_-CH_2_-CH_2_Br).

##### Acetonide **8**

From **5** (150 mg, 0.318 mmol), K_2_CO_3_ (131 mg, 0.948 mmol), 1,6-dibromohexane (240 μL, 0.934 mmol). The reaction product was purified by CC, eluting with 30% EtOAc/CH_2_Cl_2_ to give compound **8** (112 mg, 57%). ^1^H NMR: δ for the acetonide fragment: 1.40, (s, 3H, H12), 1.56 (s, 3H, H13), 3.77 (*s*, 6H, H10′, H12′), 3.82 (*s*, 3H, H11′), 6.25 (*s*, 2H, H2′, H6′), 6.38 (*s*, 1H, H3), 6.98 (*s*, 1H, H6); for the linker: 1.36–1.89 (*m*, 8H, O-CH_2_-(CH_2_)_4_-CH_2_Br), 3.99 (*m*, 6.8, 2H, O-CH_2_-(CH_2_)_4_-CH_2_Br), 4.02 (*m*, 2H, O-CH_2_-(CH_2_)_4_-CH_2_Br).

##### Acetonide **9**

From **5** (200 mg, 0.424 mmol), K_2_CO_3_ (174 mg, 1.26 mmol), α,α′-dibromo-*p*-xylene (293 mg, 1.53 mmol). The reaction product was purified by CC, eluting with 30% EtOAc/CH_2_Cl_2_ to give compound **9** (138 mg, 51%). ^1^H NMR: δ for the acetonide fragment: 1.25, (s, 3H, H12), 1.41 (s, 3H, H13), 3.75 (*s*, 6H, H10′, H12′), 3.79 (*s*, 6H, H10′, H12′), 6.24 (*s*, 2H, H2′, H6′), 6.37 (*s*, 1H, H3), 6.95 (*s*, 1H, H6); for the linker: 4.36 (*m*, 2H, O-CH_2_-C_6_H_4_-CH_2_Br), 5.09 (*d*, 6.0, 1H, O-CH_2_-C_6_H_4_-CH_2_Br), 5.19 (*d*, 6.0, 1H, O-CH_2_-C_6_H_4_-CH_2_Br), 7.10 and 7.30 (*ABq*, 8.0, 4H, O-CH_2_-C_6_H_4_-CH_2_Br).

##### MHQ **10**

From **6** (189 mg, 0.600 mmol), K_2_CO_3_ (293 mg, 2.13 mmol), 1,3-dibromopropane (231 µL, 2.13 mmol). The reaction product was purified by CC, eluting with 30% EtOAc/hexane to give compound **10** (162 mg, 52%). ^1^H NMR: δ for the MHQ fragment: 2.39, (*s*, 3H, Ac), 2.44 (*s*, 3H, Ac)_,_ 7.17 and 7.21 (*ABq*, 8.0, 2H, H2, H3), 7.32 (*dd*, 8.8 and 1.8, 1H, H7), 7.66 (*d*, 1.8, 1H, H8), 7.71 (*d*, 8.0, 1H, H5); for the linker: 1.96 (*m*, 2H, O-CH_2_-CH_2_-CH_2_Br), 3.22 (*t*, 6.8, 2H, O-CH_2_-CH_2_-CH_2_Br), 4.04 (*m*, 2H, O-CH_2_-CH_2_-CH_2_Br). IR (film, cm^−1^): 1765, 1731 (COOR). HRMS: calcd for [C_20_H_21_O_6_Br + NH_4_]^+^ 454.0870 u; found 454.0851 *m/z*.

##### MHQ **11**

From **6** (100 mg, 0.316 mmol), K_2_CO_3_ (157 mg, 1.14 mmol), 1,6-dibromohexane (294 µL, 1.14 mmol), and DMF. The reaction product was purified by CC, eluting with 40% EtOAc/hexane to give compound **11** (115 mg, 72%). ^1^H NMR: δ for the MHQ fragment: 2.37, (*s*, 3H, Ac), 2.39 (*s*, 3H, Ac)_,_ 7.17 and 7.21 (*ABq*, 8.0, 2H, H2, H3), 7.32 (*dd*, 8.8 and 1.8, 1H, H7), 7.66 (*d*, 1.8, 1H, H8), 7.71 (*d*, 8.0, 1H, H5); for the linker: 1.19–1.71 (*m*, 8H, O-CH_2_-(CH_2_)_4_-CH_2_Br), 3.28 (*t*, 7.2, 2H, O-CH_2_-(CH_2_)_4_-CH_2_Br), 3.97 (*t*, 3.8, 2H, O-CH_2_-(CH_2_)_4_-CH_2_Br). IR (film, cm^−1^): 1765, 1720 (COOR). HRMS: calcd for [C_23_H_27_O_6_Br + NH_4_]^+^ 496.1329 u; found 496.1322 *m*/*z*.

##### MHQ **12**

From **6** (121 mg, 0.316 mmol), K_2_CO_3_ (185 mg, 1.35 mmol), α,α′-dibromo-*p*-xylene (356 mg, 1.35 mmol). The reaction product that was purified by CC, eluting with 30% EtOAc/hexane to give compound **12** (87.9 mg, 43%). ^1^H NMR: δ for the MHQ fragment: 2.46, (*s*, 3H, Ac), 2.46 (*s*, 3H, Ac)_,_ 7.20 and 7.22 (*ABq*, 8.0, 2H, H2, H3), 7.38 (*dd*, 8.8 and 1.8, 1H, H7), 7.66 (*d*, 1.8, 1H, H8), 7.80 (*d*, 8.0, 1H, H5); for the linker: 4.47 (*s*, 2H, O-CH_2_-C_6_H_4_-CH_2_Br), 5.10 (*s*, 2H, O-CH_2_-C_6_H_4_-CH_2_Br). IR (film, cm^−1^): 1762, 1736 (COOR). HRMS: calcd for [C_25_H_23_O_6_Br + NH_4_]^+^ 516.1016 u; found 516.1010 *m*/*z*.

##### Compound **13**

(Route A) From **7** (236 mg, 0.400 mmol), K_2_CO_3_ (157 mg, 1.14 mmol), MHQ **6** (100 mg, 0.316 mmol) in dry DMF (2.5 mL) using the general method during 3 h. The reaction product was purified by CC on silica gel, eluting with 50% EtOAc/hexane to give conjugate **13** (138 mg, 44%). (Route B) From **10** (189 mg, 0.433 mmol), K_2_CO_3_ (165 mg, 1.2 mmol), and acetonide **5** (189 mg, 0.400 mmol) in dry DMF (2.5 mL) using the general method during 3 h. The reaction product was purified by CC on silica gel, eluting with 50% EtOAc/hexane to give conjugate **13** (234 mg, 70%). ^1^H NMR: δ for the MHQ fragment: 2.44 (*s*, 3H, Ac), 2.45 (*s*, 3H, Ac), 7.17 and 7.21 (*ABq*, 8.0, 2H, H2, H3); for the cyclolignan fragment: 1.49, (*s*, 3H, H12), 1.55 (*s*, 3H, H13), 2.17 (m, 1H, H8), 2.78 (*dd*, 4.8 and 2.0, 1H, H8′), 3.68 (*dd*, 11.2 and 4.4, 1H, H9b), 3.78 (*s*, 6H, H10′, H12′), 3.78 (*s*, 3H, H11′), 4.04 (*m*, 1H, H9a), 6.25 (*s*, 2H, H2′, H6′), 6.38 (*s*, 1H, H3), 6.97 (s, 1H, H6); for the linker: 1.80 (*m*, 2H, *MHQ*-O-CH_2_-CH_2_-CH_2_-O-*L*), 3.80 (*m*, 2H, *MHQ*-O-CH_2_-CH_2_-CH_2_-O-*L*), 4.02 (*m*, 2H, *MHQ*-O-CH_2_-CH_2_-CH_2_-O-*L*).

##### Compound **14**

(Route A) From **8** (361 mg, 0.568 mmol), K_2_CO_3_ (235 mg, 1.71 mmol), MHQ **6** (150 mg, 0.566 mmol) in dry DMF (2.5 mL) using the general method during 3 h. The reaction product was purified by CC on silica gel, eluting with 50% EtOAc/hexane to give conjugate **14** (268 mg, 54%). (Route B) From **11** (140 mg, 0.293 mmol), K_2_CO_3_ (137 mg, 0.991 mmol), acetonide **5** (137 mg, 0.290 mmol) in dry DMF (2.5 mL) using the general method during 3 h. The reaction product was purified by CC on silica gel, eluting with 10% EtOAc/CH_2_Cl_2_ to give conjugate **14** (101 mg, 41%). ^1^H NMR: δ for the MHQ fragment: 2.44 (*s*, 3H, Ac), 2.45 (*s*, 3H, Ac), 7.17 and 7.21 (*ABq*, 8.0, 2H, H2, H3); 1.49, (*s*, 3H, H12), 1.55 (*s*, 3H, H13), 2.17 (*m*, 1H, H8), 2.78 (*dd*, 4.8 and 2.0, 1H, H8′), 3.68 (*dd*, 11.2 and 4.4, 1H, H9b), 3.78 (*s,* 6H, H10′, H12′), 3.78 (*s*, 3H, H11′), 4.04 (*m*, 1H, H9a), 6.25 (*s*, 2H, H2′, H6′), 6.38 (*s*, 1H, H3), 6.97 (*s*, 1H, H6); for the linker: 1.25–1.80 (*m*, 8H, *MHQ*-O-CH_2_-(CH_2_)_4_-CH_2_-O-*L*), 3.99 (*m*, 2H, *MHQ*-O-CH_2_-(CH_2_)_4_-CH_2_-O-*L*), 4.02 (*m*, 2H, *MHQ*-CH_2_-(CH_2_)_4_-CH_2_-O-*L*).

##### Compound **15**

(Route A) From **9** (153 mg, 0.233 mmol), K_2_CO_3_ (47 mg, 0.350 mmol), MHQ **6** (73 mg, 0.230 mmol) in dry DMF (2.5 mL) using the general method during 3 h. The reaction product was purified by CC on silica gel, eluting with 5% EtOAc/CH_2_Cl_2_ to give conjugate **15** (152 mg, 51%). (Route B) From **12** (155 mg, 0.311 mmol), K_2_CO_3_ (161 mg, 1.17 mmol), acetonide **5** (154 mg, 0.326 mmol) in dry DMF (2.5 mL) using the general method during 3 h. The reaction product was purified by CC on silica gel, eluting with 30% EtOAc/CH_2_Cl_2_ to give conjugate **15** (125 mg, 42%). ^1^H NMR: δ for MHQ fragment: 2.45 (s, 6H, Ac), 7.18 and 7.22 (*ABq*, 8.0, 2H, H2, H3); for the cyclolignan fragment: 1.34, (*s*, 3H, H12), 1.51 (*s*, 3H, H13), 2.25 (*m*, 1H, H8), 2.82 (*dd*, 4.0 and 2.4, 1H, H8′), 3.63 (*dd*, 11.6 and 3.6, 1H, H9b), 3.74 (*s*, 6H, H10′, H12′), 3.79 (*s*, 3H, H11′), 3.96 (*t*, 11.6, 1H, H9a), 6.24 (*s*, 2H, H2′, H6′), 6.39 (*s*, 1H, H3), 6.95 (*s*, 1H, H6); for the linker: 5.09 (*s*, 2H, *MHQ*-O-CH_2_-C_6_H_4_-CH_2_-O-*L*), 5.19 (*s*, 2H, *MHQ*-O-CH_2_-C_6_H_4_-CH_2_-*L*), 7.10 and 7.24 (*ABq*, 7.6, 4H, *MHQ*-O-CH_2_-C_6_H_4_-CH_2_-*L*).

#### 2.1.3. General Method for the Synthesis of Hybrids Lignomonoterpenylnaphthohydroquinones (L-MHQs) **16**–**18**

The acetonide hybrids **13**–**15** were dissolved in 2:8 water/acetone solution (10 mL), and *p*-toluenesulfonic acid (10 mg, 0.05 mmol) was added and stirred at room temperature during 24 h. After this time, the acetone was concentrated under vacuum, EtOAc was added, the crude mixture was washed with brine, dried over Na_2_SO_4_, and filtered, and the organic solvent was evaporated off. The crude dihydroxyesters were submitted to Swern oxidation.

*Swern oxidation*: To a precooled (−55 °C) and stirred solution of oxalyl chloride (3 equiv.) in dry CH_2_Cl_2_ (5 mL), a solution of DMSO (6 equiv.) was added dropwise in dry CH_2_Cl_2_ (2 mL). After 5 min at −55 °C, a solution of the corresponding dihydroxyester (1 equiv.) in dry CH_2_Cl_2_ was slowly added. The mixture was stirred at the same temperature for 30 min and then triethylamine (10 equiv.) was added dropwise. The mixture was warmed to 0 °C over 1 h, quenched with water, and extracted with CH_2_Cl_2_. The organic phase was washed with 2 N HCl and saturated aqueous solutions of NaHCO_3_ and NaCl, and the solvent was evaporated off. CC of the reaction products, eluting with 20% EtOAc/CH_2_Cl_2_, gave the corresponding aldehydes.

##### Aldehyde **16**

From **13** (210 mg, 0.253 mmol) following the above general method to give aldehyde **16** (99 mg, 49%). ^1^H NMR: δ for the MHQ fragment: 2.43 (*s*, 3H, Ac), 2.45 (*s*, 3H, Ac), 7.18 and 7.21 (*ABq*, 8.0, 2H, H2, H3); for the cyclolignan fragment: 3.82 (*d*, 3.6, 1H H8′), 4.59 (*d*, 4.0, 1H, H7′), 6.21 (*s*, 2H, H2′, H6′), 6.47 (*s*, 1H, H3), 6.87 (*s*, 1H, H6), 7.33 (*s*, 1H, H7), 9.59 (*s*, 1H, H9); for the linker: 1.88 (*m*, 2H, *MHQ*-O-CH_2_-CH_2_-CH_2_-O-*L*), 3.98 (*t*, 4.0, 2H, *MHQ*-O-CH_2_-CH_2_-CH_2_-O-*L*), 4.06 (*t*, 6.0, 2H, *MHQ*-O-CH_2_-CH_2_-CH_2_-O-*L*). HRMS: calcd for [C_42_H_40_O_14_ + NH_4_]^+^ 786.2756 u; found 786.2758 *m/z*.

##### Aldehyde **17**

From **14** (465 mg, 0.534 mmol) following the above general method to give aldehyde **17** (290 mg, 67%). ^1^H NMR: δ for the MHQ fragment: 2.43 (*s*, 3H, Ac), 2.45 (*s*, 3H, Ac), 7.17 and 7.20 (*ABq*, 8.0, 2H, H2, H3); for the cyclolignan fragment: 3.97 (*m*, 1H, H8′), 4.59 (*d*, 3.2, 1H, H7′), 6.20 (*s*, 2H, H2′, H6′), 6.50 (*s*, 1H, H3), 6.87 (*s*, 1H, H6), 7.41 (*s*, 1H, H7), 9.66 (*s*, 1H, H9); for the linker: 1.20–1.54 (*m*, 8H, *MHQ*-O-CH_2_-(CH_2_)_4_-CH_2_-O-*L*), 3.97 (*m*, 2H, *MHQ*-O-CH_2_-(CH_2_)_4_-CH_2_-O-*L*), 4.02 (*t*, 6.8, 2H, *MHQ*-O-CH_2_-(CH_2_)_4_-CH_2_-O-*L*). HRMS: calcd for [C_45_H_46_O_14_ + NH_4_]^+^ 828.3225 u; found 828.3224 *m*/*z*.

##### Aldehyde **18**

From **15** (152 mg, 0.170 mmol) following the above general method to give aldehyde **18** (63 mg, 48%). ^1^H NMR: δ for the MHQ fragment: 2.44 (*s*, 3H, Ac), 2.45 (*s*, 3H, Ac), 7.18 and 7.22 (*ABq*, 8.0, 2H, H2, H3); for the cyclolignan fragment: 4.04 (*d*, 4.0, 1H, H8′), 4.58 (*d*, 4.0, 1H, H7′), 6.20 (*s*, 2H, H2′, H6′), 6.61 (*s*, 1H, H3), 6.88 (*s*, 1H, H6), 7.38 (*s*, 1H, H7), 9.59 (*s*, 1H, H9); for the linker: 5.06 (*d*, 10.8, 1H, *MHQ*-O-CH_2_-C_6_H_4_-CH_2_-O-*L*), 5.08 (*d*, 10.8, 1H, *MHQ*-O-CH_2_-C_6_H_4_-CH_2_-O-*L*), 5.09 (*s*, 1H, *MHQ*-O-CH_2_-C_6_H_4_-CH_2_-O-*L*), 7.10 and 7.24 (*ABq*, 7.6, 4H, *MHQ*-O-CH_2_-C_6_H_4_-CH_2_-O-*L*). HRMS: calcd for [C_47_H_42_O_14_ + nNH_4_]^+^ 848.2912 u; found 848.2915 *m/z*.

### 2.2. Biological Methods

**Cell lines and culture conditions**: The human colorectal adenocarcinoma (HT29, ATCC HTB-38), the human breast cancer (MCF7, ATCC HTB-22), and the human osteosarcoma (MG-63, ATCC CRL-1427) cell lines were grown in an adherent monolayer culture in Dulbecco’s Minimum Essential Medium (DMEM) for HT29 and MCF7 cells and RPMI medium for MG-63, both supplemented with 10% heat-inactivated fetal bovine serum (FBS), 1% L-glutamine, and 1% penicillin/streptomycin in a humidified atmosphere (5% CO_2_, 37 °C). All media and supplements were purchased from Sigma (St. Louis, MO, USA).

**Cytotoxic screening**: MTT (3-(4,5-dimethyl-2-thiazolyl)-2,5-diphenyl-2H-tetrazolium bromide) assay (Sigma) was employed to determine the cytotoxicity in HT-29, MCF-7, and MG-63 cells. A total of 2500 cells/well were seeded in 100 µL of media into 96-well plates (Corning Inc., Corning, NY, USA) at 37 °C in a 5% CO_2_ atmosphere. Cells were allowed for 24 h to settle and expand to near confluence. Afterward, the growing medium was replaced by fresh medium containing serial dilutions of the chemical compounds. After time exposure (72 h), the medium was replaced with fresh medium plus 10 µL/well of phosphate-buffered saline containing MTT (5 mg/mL) and the plates were further incubated for 4 h. Then, medium/MTT mixtures were replaced with DMSO (200 μL/well) as solubilization solution. Optical densities were determined at a wavelength of 570 nm (with a reference wavelength of 690 nm) by using the Synergy™ 4 Hybrid Microplate reader and analyzed using Gen5 Data Analysis software (BioTek Instruments, Winooski, VT, USA) [45].

**Flow cytometry assays for apoptosis and cell cycle assays:** A total of 200,000 cells of HT29 were seeded in 35 mm cell culture dishes (Falcon, Corning, NY, USA) and allowed to settle for 24 h. Then, chemical compounds (1 µM) were added to expose the cells for 24 h. After the incubation period, cells were detached by trypsinization and collected into flow cytometry tubes together with the supernatants. After three washing steps with PBS (1200 rpm, 3 min), cells were divided into two tubes to carry out the assays. To determine early and late apoptosis as well as cell cycle arrest, the annexin V/propidium iodide (PI) assay (ImmunoStep, S.L., Salamanca, Spain) was applied. In addition, 1× annexin-binding buffer was added to re-suspend the cells at a 1 × 10^6^ cells/mL concentration. Then, cells were systematically stained with 5 μL of the annexin-V fluorescein isothiocyanate (FITC) and 5 μL of the propidium iodide and incubated for 15 min at room temperature in the dark. After this time, 400 μL of 1× annexin-binding buffer was added. To study the cell cycle, 200 μL of Cycloscope™ Reagent (Cytognos, Salamanca, Spain) containing PI and detergent was added and cells were incubated for 10 min at room temperature in the dark. In both flow cytometry assays, data acquisition was performed in a FACS Canto II flow cytometer (Becton Dickinson Biosciences, BD, San Jose, CA, USA) using the FACS Diva software (v6.1; BD). For data analysis, the Infinicyt™ 2.0.6 software (Cytognos SL) was used.

### 2.3. Docking Methods

As described in Ref. [46], we built a model for the tubulin-dimer based on the PDB 1SA1 [47] crystal structure, for which podophyllotoxin was resolved at the colchicine site, close to the dimerization interface of the tubulin-dimer. Coordinates of the hydrogen atoms were generated with CHARMM [48] using standard protonation states for all the titratable residues. The system was placed in the center of a cubic water box that contained the protein and at least 10 Å of solvent on all sides. K^+^ and Cl^−^ ions were added to the system to account for a 0.15 M KCl concentration. Subsequently, 200 ns of molecular dynamics (MD) simulations at constant temperature (303.15 K) and pressure (1 bar) were run using NAMD [49] and the CHARMM36 force-field [50,51]. The Particle Mesh Ewald method [52] was used to account for the electrostatics of the periodic boundary conditions, and a 2 fs time step and the ShakeH algorithm were used. Parameters for podophyllotoxin were assigned using ParamChem [53,54].

We extracted the coordinates of the protein chain for 5 representative frames of the MD simulation, which were used as templates for the docking step, carried out using Autodock-Vina [55]. Docking energies reported in the text correspond to the largest docking energy. When podophyllotoxin was docked, the most stable pose was always equivalent to that in the original frame, which validates the docking procedure.

## 3. Results

### 3.1. Synthesis of the New Lignohydroquinones

**Scheme 1:**
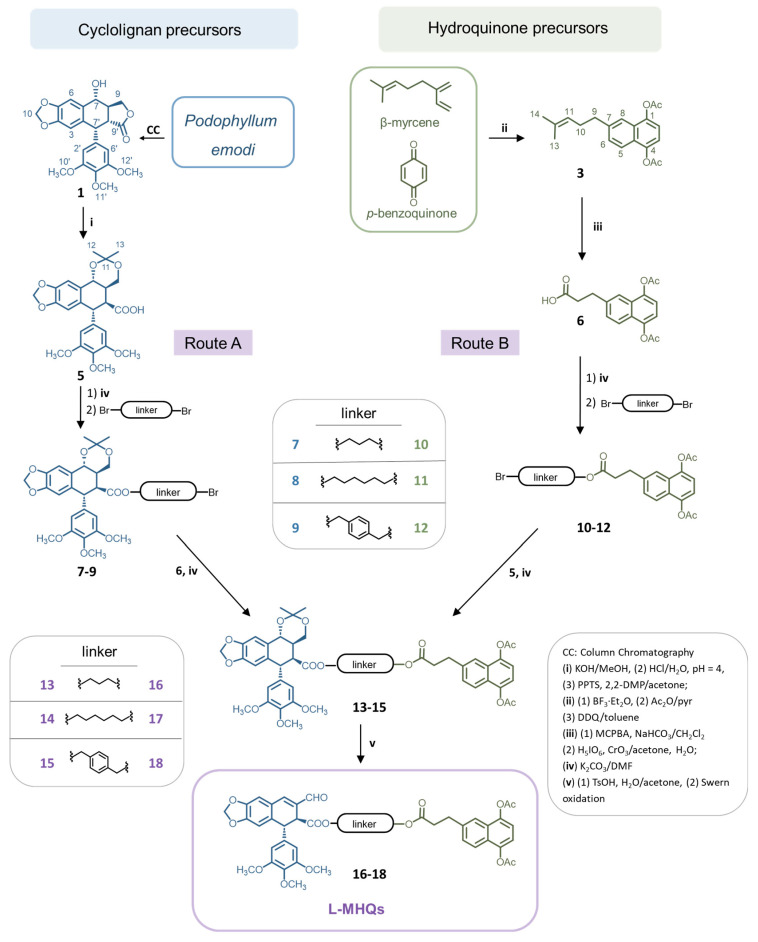
Synthesis of the new L-MHQs **16**–**18**. PPTS: pyridinium *p*-toluenesulfonate. DMP: 2,2-dimethoxypropane. Pyr: pyridine. DDQ: 2,3-dichloro-5,6-dicyano-1,4-benzoquinone. MCPBA: *m*-chloroperbenzoic acid. DMF: dimethylformamide. TsOH: *p*-toluenesulfonic acid.

The first step in the synthesis of final L-MHQs is the obtaining of precursors provided with the proper functional groups for the attachment of the chemical linkers (Scheme 1).

For the cyclolignan fragment of the family, the intermediate **5** was easily obtained from podophyllotoxin, **1**, isolated from the rhizome resin of *Podophyllum emodi* as previously described by us [35]. The opening of the lactone ring was followed by the formation of the acetonide provided **5**, which had an epimerized free carboxylic acid, that enabled the subsequent ester formation with the corresponding α,ω-dibromolinkers to yield cyclolignans **7**–**9** (Scheme 1).

The MHQ precursor **3** was obtained from the naturally occurring monoterpenoid β-myrcene, which is commercially available, by cycloaddition with the *p*-benzoquinone followed by acetylation and aromatization as previously described [44]. Subsequent oxidative cleavage of the side-chain double bond of **3** led to the MHQ **6** that had the carboxylic acid necessary for the reaction with the linkers [43]. Aliphatic and aromatic spacers were proposed to join the two moieties of the hybrid and to analyze the influence on the bioactivity of this family. From cyclolignan **5** and MHQ **6**, it is already possible to obtain the L-MHQs proposed in the objectives of the work. As both precursors were obtained with a good feasibility, two synthetic routes were proposed to obtain the L-MHQs depending on whether the linkers are first attached to one or to the other precursor (route A or B, Scheme 1). For both precursors, the same reaction conditions (K_2_CO_3_ and DMF) optimized in previous work were used [38] to yield compounds **7**–**9** from **5** and derivates **10**–**12** from **6**. Then, compounds **13**–**15** were obtained either from **7**–**9** (route A) or from **10**–**12** (route B). Yield differences were observed depending on the linker and the route followed. We observed that the linkers that induce less flexibility or more rigidity later to the molecule (short aliphatic chain and aromatic ring) should be joined first to the lignan fragment (route A). Meanwhile, for the longest and more flexible aliphatic chain, better yield was obtained, starting the synthesis by linking the spacer to the naphthohydroquinonic rest (route B) (see Section 2.1.2 in Section 2).

Based on our previous experience [39], the last step in the synthesis of the hybrids is the transformation of the cyclolignan moiety of **13**–**15** into the corresponding C-9 α,β-unsaturated aldehyde by hydrolysis of the acetonide followed by the oxidation under Swern conditions, which led to **16**–**18**, L-MHQ analogs to the podophyllic aldehyde.

### 3.2. Biological Assays

Both fragments of the hybrids differ not only in their chemical structure, but also in their biological activity. Hydroquinonic and quinonic scaffolds are known to trigger intracellular oxidative stress resulting in a rapid induction of apoptosis [56]. By contrast, cyclolignans derived from podophyllotoxin and non-lactonic derivates were demonstrated to be potent antimitotic agents requiring longer drug exposure times to detect their potential as cell death inducers [37]. This duality was explored in the new hybrids presented here, evaluating the compounds in different tumor cell lines, through various biological approaches and with different exposure times of cells to the compounds. In previous reports, final hybrids have been tested together with the corresponding starting synthetic materials [38,39]. In this case, representing each component of the hybrid, the drug podophyllotoxin, **1**, the lead compound for this work podophyllic aldehyde, **2**, and the precursor MHQ, **3,** were also included in the assays for comparison with the final hybrids.

#### 3.2.1. Cytotoxicity Evaluation

The first step for determining the biological potential of our novel compounds was the evaluation of the in vitro cytotoxicity on three different tumor cell lines: MG-63 (osteosarcoma), MCF-7 (breast adenocarcinoma), and HT-29 (colon adenocarcinoma). Cells were tested at 24 and 72 h of drug exposure time to evaluate differences in cytotoxicity. The IC_50_ values found for the precursors and the hybrids are shown in Table 1 (Statistical analysis provided in Table S1) where the above-mentioned differences in the effects of both types of hybrid fragments are clearly observed. Thus, the MHQ **3** showed cytotoxicity at the µM level independently of the exposure time on the three cell lines, while the cyclolignans **1** and **2** showed less cytotoxicity and higher IC_50_ values at 24 h than at 72 h incubation time. Both were practically inactive (IC_50_ > 100 µM) against MCF-7 and HT-29 at 24 h, contrasting with being the most potent at the longest exposure time with IC_50_ values at the nM level. The other MHQs **10**–**12** were also evaluated at 72 h of incubation (Table S1) and they showed similar cytotoxicity values as **3**, in the µM level.

Regarding **16**–**18**, they were cytotoxic at the µM level or below after both exposure times, with the increment in cytotoxicity observed at 24 h of exposure time being interesting in comparison with **1** and **2** and reaching the nM level at the end of the cytotoxicity study.

Considering each tumor cell line separately, differences in sensitivity and behavior were also observed. MCF-7 cells resulted as the least sensitive to the L-MHQs, with all the IC_50_ values at the µM range at any time of incubation. In the case of MG-63 cells, L-MHQ **16** with the shortest spacer showed no differences between both incubation times, while L-MHQs **17** and **18**, with longer and more rigid spacers, respectively, increased their cytotoxicity at 72 h of incubation. Both also presented IC_50_ values almost 10 times lower than that of hybrid **16** at long incubation time, indicating that the spacer influences the final activity, with the long and the rigid spacers being better than the short one for this tumoral cell line at long exposure time (0.260 and 0.292 for **17** and **18**, respectively, *vs* 2.26 µM for **16**).

This trend was maintained for HT-29 cells, where the most interesting values of cytotoxicity were observed at the longest incubation time. In particular, **18**, with the aromatic spacer on its structure, obtained an IC_50_ value comparable to the cyclolignan precursors (66.4 and 28.3 nM for **1** and **2,** respectively**,** and 40.5 nM for **18**), being several times more potent against this cell line than the other two. Therefore, it can be said that **18** not only maintained cytotoxicity against HT-29 but also improved its selectivity against this line. The selectivity observed for **18** was in accordance with our previous results with other cyclolignan hybrids as the lignopurines [39]. These interesting results in cytotoxicity and selectivity prompted us to deepen the implied mechanism of action of the novel conjugates.

#### 3.2.2. Flow Cytometry Assays

**Figure 3:**
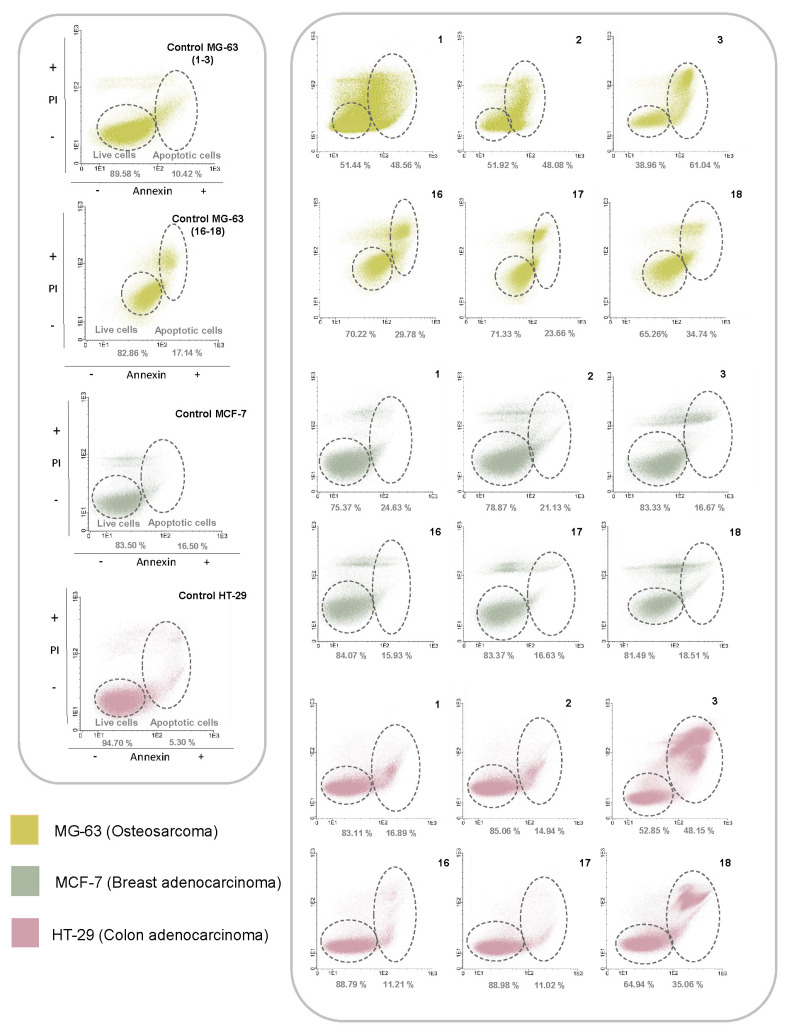
Apoptosis assays results observed by flow cytometry for the precursors **1**–**3** and the hybrids L-MHQs **16**–**18** in MG-63 (osteosarcoma), MCF-7 (breast cancer), HT-29 (colon adenocarcinoma) cells at 24 h of incubation at 1 µM. Control with vehicle (DMSO, <0.001%).

**Figure 4:**
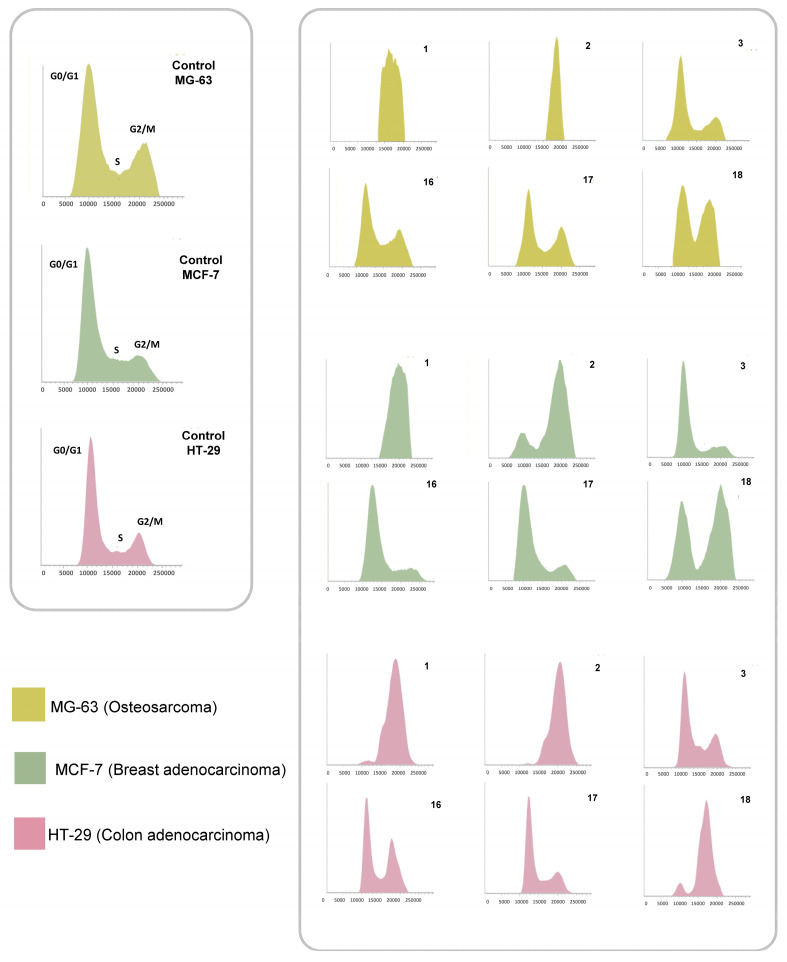
Effect of the precursors **1**–**3** and the hybrids **16**–**18** on DNA-ploidy determined by flow cytometric analysis to determine cell cycle phase. The cells were incubated with 1 μM of the indicated compound and control for each line with the vehicle (DMSO, <0.001%) for 24 h.

To analyze the cell death mechanisms involved in final hybrids and precursors, an extensive flow cytometry study was carried out. Several interesting conclusions have been drawn from the analysis of apoptosis induction and cell cycle arrest processes (Figure 3 and Figure 4).

Precursors (**1**, **2**, and **3**) and L-MHQs **16**–**18** were evaluated at 1 μM concentration in the three cells lines studied at 24 h of incubation. This assay had let us assess interesting insights into the type of cell death behavior induced by our compounds. According to our previous studies with the precursors separately, cyclolignans and terpenylnaphthohydroquinones had a different way of promoting apoptosis that can be observed in annexin/PI determination by flow cytometry [38]. The results are shown in Figure 3 and in Table S2.

Considering the type of tumor cell line, MG-63 seemed to be the type of cell where a stronger apoptotic effect appeared at this time of incubation. Particularly, precursors presented the highest values, but the effect was not reflected in final hybrids. In this line, it is also worth observing the difference in the propidium iodide (IP) ratio (PI−/PI+) that allows the differentiation between early apoptosis (higher IP-) and late apoptosis (higher IP+). Considering this relationship, the cyclolignan precursors led to a higher population of cells in early apoptosis (PI−) than the MHQ precursor, which had a higher population of PI+ cells. The other two cell lines studied showed slight apoptotic responses. This agreed with the observed cytotoxic results for **1** and **2**, which did not present cytotoxicity at 24 h of incubation (Table 1). Particularly, in the HT-29 line, **1** and **2** presented a slight apoptotic response, of an early character too, as occurred in the MG-63 line. In addition, the apoptotic response of the MHQ **3** in HT-29 line was striking, with a marked late apoptosis (PI+). Regarding the apoptotic response for the hybrids, it was especially intense for L-MHQ **18**, which showed an increase in annexin+ cells and also in the PI−/PI+ ratio, presenting a behavior more similar to the MHQ precursor than to cyclolignans.

Flow cytometry was also used to determine the effect of the compounds on the cell cycle (Figure 4 and Table S3). In this matter, it is interesting to observe the cell population in the G2/M phase as cyclolignans, inhibitors of tubulin polymerization, arrest cell division at this mitosis phase.

As expected, cyclolignans **1** and **2** blocked the cell cycle almost completely in the three lines studied, whereas MHQ **3** had no effect on the cell cycle. For the hybrids, the results varied considering the chemical characteristics of the spacers. In general, L-MHQs with aliphatic spacers (**16** and **17**) did not show a cell cycle blockade at this incubation time, except for **16**, which exhibited a slight arrest in HT-29 cells. As already observed in the cytotoxicity tests, hybrid **18** showed the best results. This compound was able to block the cycle in the three lines studied: partial blockade in MG-63 and MCF-7 cells and total arrest in HT-29 line. These results together with its high cytotoxicity in HT-29 cells at long incubation times prompted us to further investigate L-MHQ **18** to elucidate whether the cell death mechanisms involved in both independent moieties may be observed in this hybrid. Thus, the study was carried out at different concentrations and at different incubation times in HT-29 cells. The differences in cell death between precursors and L-MHQ **18** indicated a combination of the activities of both precursors in the final hybrid.

**Figure 5:**
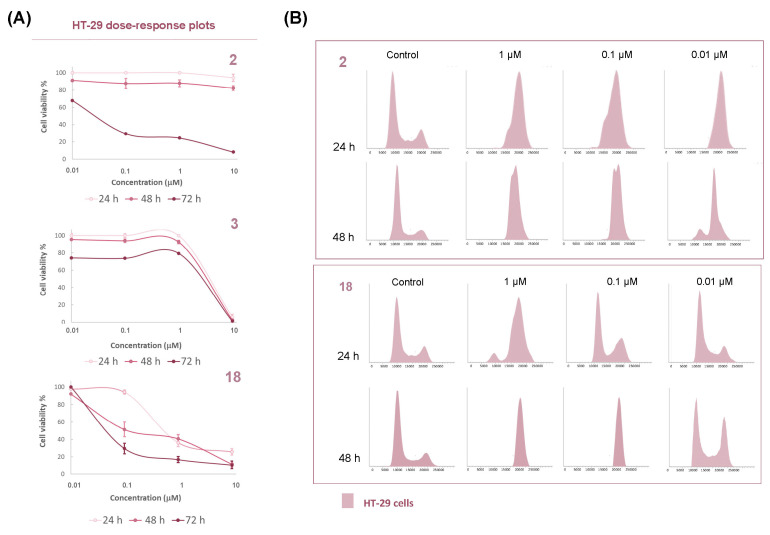
(**A**) Dose–response plots of the precursors **2** and **3** and the hybrid **18** at different times of incubation (24, 48, and 72 h) and concentrations (0.01–10 µM) in HT-29 cells. (**B**) Flow cytometry plots of the effect of **2** and **18** at different times of incubation (24 and 48 h) and concentrations (1–0.01 µM) in HT-29 cells.

Starting with the dose–response plots of the compounds at different incubation times (Figure 5A), it can be observed how hybrid **18** showed cytotoxicity from the earliest incubation time, as occurred with precursor **3**. In comparison, precursor **2** only showed cytotoxicity at the longest incubation time. That early cell death pattern observed for L-MHQ **18** comparable to that of MHQ **3** and not shown by cyclolignan **2** illustrates how the MHQ part is playing a role in the cell death mechanism of the L-MHQ **18**. Additionally, a gradual evolution can be seen for **18**, which induced higher cytotoxic effect as the incubation time increased unlike what was observed in the precursors. Thus, podophyllic aldehyde, **2**, only showed cytotoxicity at the last incubation time and MHQ **3** did not present a greater response beyond 24 h of incubation. In order to correlate the final cytotoxicity of L-MHQ **18** with the antimitotic activity of cyclolignans, such as **2**, the HT-29 cell cycle blockade was studied up to a dilution of 10 nM (level of IC_50_ at 72 h of incubation) to corroborate that this is the underlying mechanism that promotes the final cytotoxicity (Figure 5B and Table S4).

**Figure 6:**
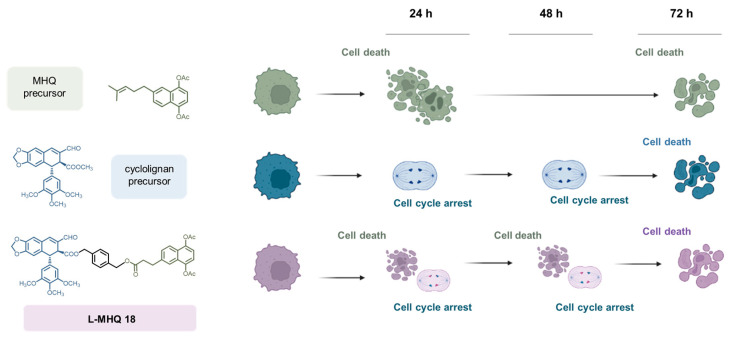
Schematic representation of cell death progression over the incubation time of the precursors **2** and **3** and the L-MHQ **18** in HT-29 cell line.

In this case, L-MHQ **18** induced cell cycle arrest at all dilutions evaluated, including the lowest dilution, as occurred with podophyllic aldehyde, **2**. These results, along with the previously observed apoptosis patterns (Figure 3) and cell cycle trends (Figure 4), suggested the involvement of dual mechanisms of cell death progression in the final cytotoxicity of the hybrid **18** (Figure 6): first, showing cell death at an early time of incubation (as the MHQ precursor **3**) and reaching the nM level at the final time of incubation (as the cyclolignan precursor **2**). This progression is illustrated in Figure 6.

### 3.3. Molecular Docking Studies

**Figure 7:**
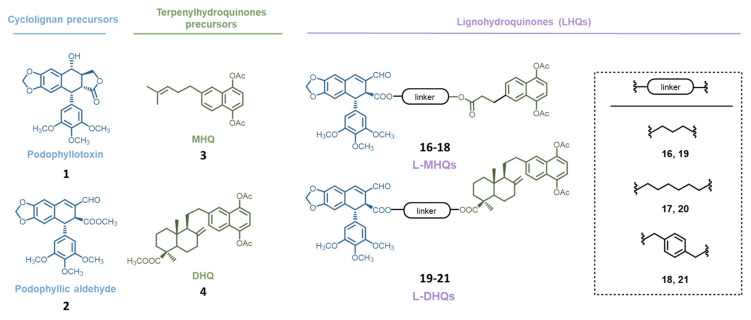
Structures of the precursors and hybrids included in the molecular dynamics and docking calculations.

Once the cell death mechanism of the hybrids was elucidated, we attempted to correlate their activity to the stability of the L-MHQ-tubulin complexes calculated through a combination of Molecular Dynamics (MD) and docking calculations (see Section 2.3 for details). We also calculated the docking energies for the previously synthesized L-DHQs **19**–**21** (Figure 7) mentioned at the chemistry design section as they also presented good cell cycle arrest values [38], comparable to those of the cyclolignan precursors.

This comparison would allow us to correlate the observed biological effects with the size of the terpenylhydroquinone attached to the C-9′ position of the cyclolignan. In addition to precursors **1** and **2** and MHQ **3** tested in this work, DHQ **4** was also included in this part of the study (Figure 7).

Simulations were carried out on the PDB 1SA1 crystal structure, in which tubulin is co-crystallized in the presence of podophyllotoxin, and docking energies are displayed in Table 2. We observed that all docking energies are similar, with most of them below −9 kcal/mol. In particular, the most stable complexes were obtained for two of the novel L-MHQs (−10.4 and −10.5 kcal/mol), suggesting that these compounds were able to prevent the α,β-heterodimer formation and the subsequent tubulin polymerization toward microtubules more successfully than the lead compound **2**. As expected, lower binding energies were obtained for the precursors **3** and **4**, although the value found for **4** almost reached that of podophyllotoxin, **1** (−9.40 vs. −9.50 kcal/mol).

According to the results of both types of L-HQs, a correlation between the IC_50_ cytotoxicity values and binding energies can be detected (Table 2). In general, the previous L-DHQs showed lower cytotoxicity, with values above the µM level (−log IC_50_ < 6), while the L-MHQs were more cytotoxic, with values closer to the nM level. This trend was also observed in the binding energies, as the most stable ligand-tubulin complexes were found for the L-MHQs presented in this report, improving the values of the precursors.

**Figure 8:**
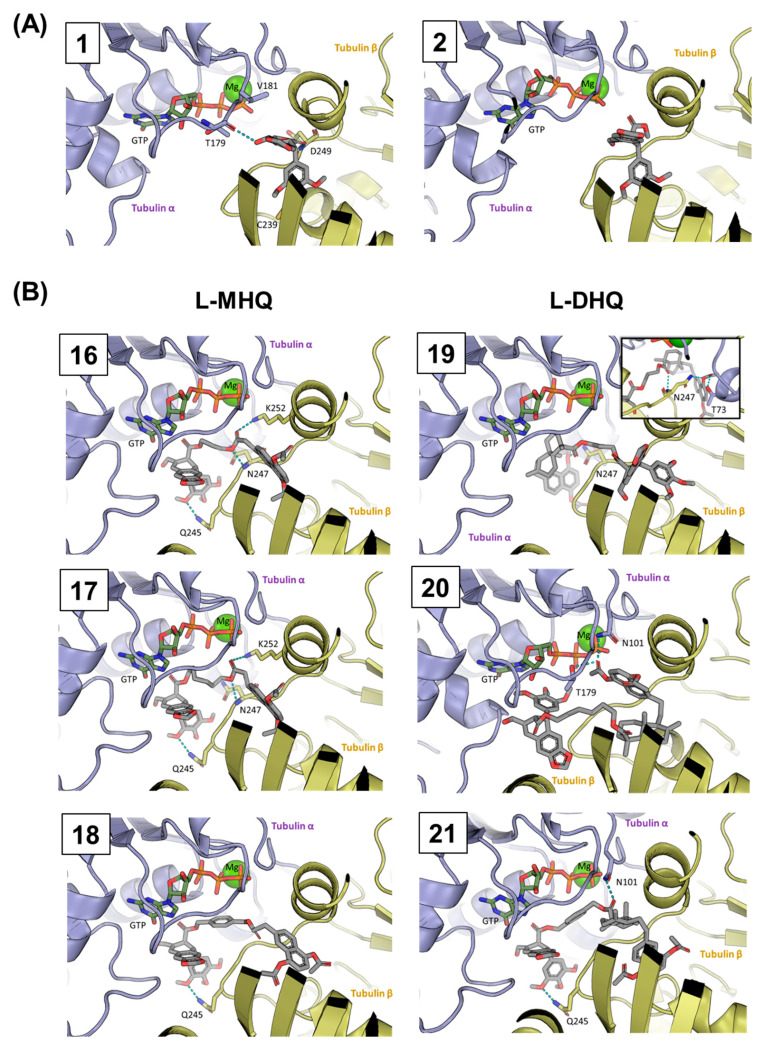
Docking complexes in the colchicine binding site of tubulin for: (**A**) precursors **1** and **2** and (**B**) L-MHQs **16**–**18** and L-DHQs **19**–**21**. GTP, the inhibitors, and those residues that interact with the inhibitors via hydrogen bonds are shown atomistically. The two protomers α- and β-tubulin are shown in purple and yellow, respectively.

Considering the influence of the linker used, the L-HQs with the aromatic linker (**18** and **21**) presented better cytotoxicity values as well as the lowest interaction energies within each series, even though the difference was not so remarkable between the series. Finally, it should be noted that the best binding energy was presented by **18**, improving considerably the energy with respect to podophyllotoxin, **1**, and podophyllic aldehyde, **2** (−10.5, −9.5, and −8.5 kcal/mol, respectively). In addition, as can be seen in Table 1, the L-MHQ **18** also presented the lowest IC_50_ values. Among the L-HQs prepared, it is noteworthy that the two compounds with the aromatic linker, **18** and **21**, achieved cell cycle arrest values similar to those of the cyclolignan precursors (Figure 4 [38]). The other L-HQs from both series did not show a remarkable cell cycle blockade and only **19** showed a partial arrest [38]. The calculated spatial arrangement of ligands in the tubulin active site was also analyzed to discern how the cyclolignan and hydroquinone moieties can contribute to the inhibition of tubulin polymerization (Figure 8). It is well known that podophyllotoxin interacts with tubulin at the colchicine binding site as other related cyclolignans do (Figure 8). This site is embedded in β-tubulin, close to the interface with α-tubulin where the GTP binding site is located. Therefore, the inclusion of molecules at this site could enable ligand interactions with some amino acids of the α-tubulin strand and, thus, prevent microtubule formation by inhibiting the polymerization of the α- and β-tubulin chains.

Podophyllotoxin, **1**, fits into the β-tubulin strand, placing the trimethoxyphenyl residue in the inner part of the pocket while the four fused rings are located closer to the interface of the two protomers (Figure 8A). This arrangement provided an interaction of **1** with both α-tubulin (T179) and β-tubulin (D249). A similar disposition is adopted by **2** (Figure 8A) as expected from their structural similarity. Terpenylhydroquinones have not been described as binding ligands to the colchicine binding site but, in this case, spatial simulations were also performed with **3** and **4** to determine which disposition within the colchicine pocket they may preferentially adopt. In this case, both the terpenic rest and hydroquinonic core of these molecules fit into the β-tubulin gap. However, the interaction of the two molecules differed in how the two structural units were arranged. While the naphthalene system of **3** was placed in a plane similar to the podophyllotoxin polycyclic system, in **4**, the diterpenoid rest occupied that pocket (Figure S1).

The most striking results were observed when studying the accommodation of L-HQs (Figure 8B). Due to its size, L-HQs did not fit entirely into the β-strand binding site, and some parts of the conjugates reached the dimerization interface and the α-strand. All the linkers occupied a critical position in the dimerization zone, determining the final disposition of the two hybrid components. Despite what might be expected, most L-HQs accommodated the MHQ or the DHQ fragments into the β-tubulin colchicine binding site instead of the cyclolignan rest. Only L-DHQ **19** was placed within the colchicine site by locating the cyclolignan core within the colchicine binding site, but its disposition differed from the precursor’s arrangement.

Comparing the L-MHQs, it can be seen that the cyclolignan rest of the three hybrids adopted a similar disposition, with the same interaction on β-tubulin through Q245 (Figure 8B). Attending to the linker influence in L-MHQs, the two hybrids with aliphatic linkers (**16** and **17**) adopted a similar accommodation of the hydroquinone rest, independently of the linker length. Both compounds adopted an arrangement that allowed them to interact with β-tubulin through a different area of the protein (K252) than podophyllotoxin, **1**. In contrast, the aromatic linker in the hybrid **18** caused the MHQ rest to adopt a different arrangement into the β-tubulin, more similar to that observed with the cyclolignan moiety in precursors **1** and **2**.

Results obtained for L-DHQs varied more with respect to the linker (Figure 8B). In this series, both aliphatic linkers provided opposite dispositions. As previously commented, L-DHQ **19** is the only hybrid for which the cyclolignan moiety was located in the β-tubulin pocket, while the DHQ moiety was placed in the same space as the cyclolignan in the other hybrids, but without interacting with the Q245 β-sheet residue. Moreover, L-DHQ **20** presented a conspicuous disposition due the length and flexibility of the linker, and the arrangement of both the cyclolignan and DHQ fragments did not correspond to any arrangement observed for the other L-HQs. In this hybrid, the disposition of the cyclolignan residue did not correspond to those observed in the precursors or in the other L-HQs that accommodated the hydroquinone residue into the β-tubulin. In this case, the trimethoxyphenyl group is disposed toward the α-tubulin and the DHQ decalin is placed in the β-tubulin pocket. Finally, the hydroquinone part interacted with α-tubulin near the GTP site.

For L-DHQ **21**, with the aromatic linker, the arrangement of the cyclolignan was like that adopted in the L-MHQs and with the same Q245 interaction. In this case, the linker occupied a similar location to the one it occupied in its L-MHQ homolog **18**, with a slight difference in its orientation between the two tubulin subunits.

Considering that **18** and **21** are the only hybrids capable of promoting a practically complete cell cycle arrest in vitro, this ability can be correlated with the presence of the rigid linker and the hybrid-tubulin interactions conditioned by it, particularly inside the colchicine binding pocket. Looking at the hydroquinone residues of **18** and **21**, both compounds interacted in the same way with β-tubulin and arranged the hydroquinone core to occupy the inner part of the colchicine pocket, which is also considered critical for the inhibition of tubulin polymerization. In the case of L-MHQs **16** and **17**, while the trimethoxyphenyl residues were arranged in the same disposition as **18**, the hydroquinone residues did not occupy the entire inner zone of the colchicine binding site, remaining closer to the interface between the protomers. The other two L-DHQs **19** and **20**, which also failed to block the cell cycle in vitro, also did not appear to occupy the colchicine pocket satisfactorily, either by the cyclolignan residue in compound **19** or by the decalin in compound **20**.

## 4. Discussion

Natural products continue being a good starting point for antitumor drug discovery. In this report, two different natural-derived compounds, cyclolignans and terpenylnaphthoquinones, were conjugated with good results both from the chemical and bioactivity points of view.

On one hand, the opening of the lactone followed by the attachment of a large substituent at the cyclolignan C-9′ position was demonstrated to be a suitable strategy for the synthesis of new podophyllotoxin-derived hybrids. Apart from our previous reports [38,39], several examples of non-lactonic cyclolignans with different substituents attached at the C-9′ position can be found in the literature [57,58,59]. However, our strategy of including an additional α,β-unsaturated aldehyde in the C-9 position significantly increased the cytotoxic potency of our compounds in comparison with the cytotoxic effect of the other reported derivatives. Thus, our conjugates showed IC_50_ values in the nM level while the other compounds reach only the µM level. In addition to the high cytotoxicity displayed by the tested compounds bearing the podophyllic aldehyde-derived fragment, a blockade of the cell division at the G2/M phase of mitosis was observed for some of them. In the present study, we demonstrated that this effect was related to tubulin polymerization inhibition and can be combined with further cytotoxic effects such as the redox potential of hydroquinone scaffolds.

On the other hand, and compared with the L-DHQs previously prepared by us bearing a large terpene residue, the approach of reducing the size of the terpene fragment carried out in this study for the second hybrid component has demonstrated that the 1,4-diacetoxynaphthalene residue was the pharmacophoric group responsible for the shortest incubation time required for L-MHQs to produce cytotoxicity. This dual cytotoxic effect was clearly evident in L-MHQ **18**, which was the most potent derivative. It reached both the cytotoxicity level of the podophyllic aldehyde, **2**, and the cytotoxicity at short incubation times of the MHQ fragment, improving the cytotoxic properties of the components separately.

Although recent studies have provided interesting results that corroborate the potential of podophyllotoxin hybridization with other natural compounds [60,61,62,63], those studies focused mainly on the junction through the C-7 position keeping the lactone ring and on the quantitative improvement of cytotoxicity with respect to the reference compound. In contrast, our study was conducted with non-lactonic cyclolignans considering different linkers and tumor lines, with the aim of analyzing not only the potency but also the selectivity against several tumor cell lines. Regarding the spacers, the aromatic one provided better cytotoxic results than the aliphatic ones, as it was also observed previously for lignopurines and other lignohydroquinones [38,39].

Furthermore, the highest antitumor potency and combined cytotoxic properties were achieved in the HT-29 colon carcinoma cell line compared to the two other tumor cell lines tested. These results are in agreement with previous studies of related non-lactonic cyclolignans, either with the α,β-unsaturated aldehyde [39] or with other electrophilic groups at the C-9 position [37]. Tubulin molecular docking studies agreed with the cytotoxic effect, confirming that the L-MHQ **18** arrangement successfully reached the inner part of the colchicine binding site in tubulin. Even if the disposition of the trimethoxyphenyl rest of cyclolignan in L-MHQ **18** did not arrange as podophyllotoxin, **1,** or podophyllic aldehyde, **2**, it seems that this disposition can be correlated with a successful inhibition of tubulin polymerization. Both compounds, L-MHQ **18** and compound **21**, which was the most potent of the series of L-DHQs [38], are arranged in the same way at the interface of the two tubulin chains. These results also provide a new perspective in hybrid design for non-lactonic cyclolignans, corroborating that the C-9′ position can incorporate different scaffolds and maintain the potent antimitotic effect of podophyllotoxin-related cyclolignans.

## 5. Conclusions

A new family of hybrids derived from natural compounds has been synthesized, merging chemical and biological features of both natural starting materials, cyclolignans and terpenylhydroquinones. The combination of both components has enhanced the cytotoxic profile of the hybrids, particularly if they are attached by an aromatic linker, as occurred for L-MHQ **18**, which showed nM cytotoxicity potency at short incubation times with a selectivity against HT-29 colorectal cancer cells.

The results of this study prove the validity of the applied hybridization methodology, encouraging further research on non-lactonic cyclolignans related to podophyllic aldehyde and also to explore other hybridization possibilities focused on the discovery and development of new antitumor drugs.

## Figures and Tables

**Table 1:** Cytotoxicity values (IC_50_, µM) of the precursors **1**–**3** and final hybrids (L-MHQs **16**–**18**).

	IC_50_ (µM)					
Compound	MG-63		MCF-7		HT-29	
	24 h	72 h	24 h	72 h	24 h	72 h
**1**	18.3	0.00707	>100	0.0284	>100	0.0664
**2**	1.23	0.0663	>100	0.477	>100	0.0283
**3**	1.11	4.23	8.23	4.93	4.33	8.51
**16**	2.80	2.26	1.38	2.33	4.77	0.834
**17**	2.70	0.260	8.81	2.34	4.16	0.652
**18**	0.752	0.292	13.1	1.78	4.12	0.0405

**Table 2:** Docking energies (−ΔG, kcal/mol) calculated for the interaction with tubulin, IC_50_ cytotoxic values expressed as −log IC_50_ for precursors and both series of hybrids.

Compounds		−ΔG (kcal/mol)		−log IC_50_	
			MG-63	MCF-7	HT-29
Cyclolignans	**1**	9.50	8.15	7.55	7.18
	**2**	8.50	7.22	6.32	7.54
Terpenylhydroquinones	**3**	9.00	5.08	5.95	5.36
	**4**	9.40	6.85	7.82	7.74
L-MHQs	**16**	10.40	5.65	5.63	6.08
	**17**	9.90	6.59	5.63	6.19
	**18**	10.50	6.54	5.75	7.39
L-DHQs	**19**	9.00	5.65	5.66	5.82
	**20**	10.10	5.69	6.00	5.72
	**21**	10.10	6.82	5.99	5.84

## Data Availability

Data sharing is not applicable to this article.

## Supplementary Materials

The following supporting information can be downloaded at: https://www.mdpi.com/article/10.3390/pharmaceutics15030886/s1.

## Abbreviations

L-HQ: lignohydroquinone; MHQ: monoterpenylhydroquinone; L-MHQ: lignomonoterpenylnaphthohydroquinone. DHQ: diterpenylnaphthohydroquinone. L-DTQ: lignoditerpenylnaphthohydroquinone. PI: propidium iodide. MD: Molecular Dynamics.
