# Supplementary material for: Improving Properties of Podophyllic Aldehyde-Derived Cyclolignans: Design, Synthesis and Evaluation of Novel Lignohydroquinones, Dual-Selective Hybrids against Colorectal Cancer Cells
Source: Pharmaceutics. 2023 Mar 9;15(3):886. doi: 10.3390/pharmaceutics15030886 (PMC10053213; doi:10.3390/pharmaceutics15030886)
Supplement: Supplementary file 1 [file pharmaceutics-15-00886-s001.zip › pharmaceutics-2242229-supplementary.pdf]

Table S1: Cytotoxicity (IC_{50}. μM ± SD) of the precursors (**1-3**) and synthesized compound (**10-12** and **16-17**) at 24 and 72 h of incubation.

Time incubation	Compound	MG-63	MCF-7	HT-29
24 h	**1**	18.3 ± 1.4	>100	0.066 ± 0.002
	**2**	1.23 ± 0.06	>100	0.028 ± 0.006
	**5**	1.11 ± 0.18	8.23 ± 0.05	4.33 ± 1.73
	**15**	2.8 ± 0.06	1.38 ± 0.14	4.77 ± 0.20
	**16**	2.7 ± 0.04	8.81± 0.21	4.16 ± 0.15
	**17**	0.75 ± 0.12	13.09 ± 0.04	4.12 ± 0.05
72 h	**1**	0.028 ± 0.003	0.007 ± 0.005	>100
	**2**	0.48 ± 0.03	0.06 ±0.02	>100
	**5**	4.93 ± 0.76	4.93 ± 0.76	8.51 ± 0.01
	**9**	1.82 ± 0.18	3.97 ± 0.23	0.26 ± 0.07
	**10**	0.55 ± 0.35	0.87 ± 0.13	0.88 ± 0.41
	**11**	4.45 ± 3.68	4.86 ± 0.98	2.90 ± 0.84
	**15**	2.33 ± 0.78	2.26 ± 0.88	0.83 ± 0.25
	**16**	2.34 ± 0.25	0.26 ± 0.08	0.65 ± 0.02
	**17**	1.78 ± 0.20	0.29 ± 0.17	0.041 ± 0.005

Table S2: % apoptotic cells (ratios Annexin +/- and PI +/-) at 24 h of incubation

Cell line	Compound	Concentration	Annexin - (%)	Annexin + Total (%)	Annexin + PI- (%)	Annexin + PI+ (%)
**MCF-7**	**Control**		83.50	16.50	17.76	86.24
	**1**	1 µM	75.36	24.64	91.33	8.67
	**2**	1 µM	78.86	21.14	89.04	10.96
	**3**	1 µM	38.69	61.04	52.87	47.13
	**16**	1 µM	84.07	15.93	56.33	43.47
	**17**	1 µM	83.37	16.63	40.81	59.19
	**18**	1 µM	81.49	18.51	20.03	79.97
**MG-63**	**Control**		89.58	10.42	33.32	66.68
	**1**	1 µM	51.44	48.56	64.62	35.38
	**2**	1 µM	51.92	48.08	81.29	18.71
	**3**	1 µM	38.69	61.04	52.87	47.13
	**16**	1 µM	70.22	29.78	93.12	6.88
	**17**	1 µM	71.33	28.66	62.35	37.65
	**18**	1 µM	65.26	34.74	83.25	16.75
**HT-29**	**Control**		94.70	5.30	22.55	77.45
	**1**	1 µM	94.70	5.30	22.55	77.45
	**2**	1 µM	83.11	16.89	79.60	20.40
		0.1 µM	87.47	12.53	92.01	7.99
		0.01 µM	86.97	13.04	-	-
	**3**	1 µM	52.85	48.15	11.41	88.59
		0.1 µM	93.60	6.40	-	-
		0.01 µM	94.10	5.90	-	-
	**16**	1 µM	88.79	11.21	56.33	43.47
	**17**	1 µM	88.98	11.02	40.81	59.19
	**18**	1 µM	64.94	35.06	13.02	86.98
		0.1 µM	89.29	13.71	-	-
		0.01 µM	94.35	5.65	-	-

Table S3: % apoptotic cells (ratios Annexin) at 48 h of incubation in HT-29 cell line.

**Compound**	Concentration	**Annexin - (%)**	**Annexin + (%)**
**Control**		90.90	10.10
**2**	1 μM	87.92	12.08
	0.1 μM	87.58	12.42
	0.01 μM	91.20	8.80
**Control**		86.43	13.50
**3**	1 μM	87.92	12.08
	0.1 μM	89.35	10.65
	0.01 μM	91.32	8.68
**18**	1 μM	43.67	56.30
	0.1 μM	44.79	55.21
	0.01 μM	71.14	27.86

Table S4: results (%) of cells in each cell cyle phase at 24 of incubation

	MG63			MCF7			HT-29		
	G0/G1	S	G2/M	G0/G1	S	G2/M	G0/G1	S	G2/M
**1**	0	0	100	0	0	100	3.1	26.02	70.85
**2**	0	0	100	3.76	1.80	94.63	2.15	5.62	92.29
**3**	55.04	11.64	34.83	73.09	7.02	19.89	59.99	10.92	29.10
**16**	64.07	13.90	24.29	74.03	7.85	18.13	49.45	10.48	40.07
**17**	67.11	13.24	21.33	76.16	8.80	15.05	62.72	14.80	22.49
**18**	43.16	18.41	47.03	35.00	9.38	55.63	10.42	5.03	84.55
**Control**	56.49	14.74	28.15	62.65	14.87	23.93	61.92	13.02	25.03

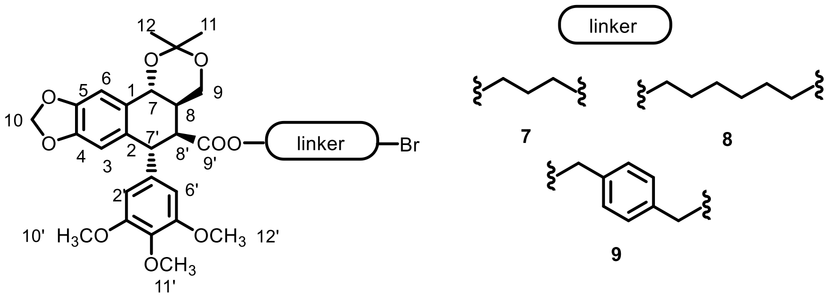

Table S5: ^{1}H NMR for compound **7-9**, δ in ppm (*J* in Hz)

	H	7	8	9
Cyclolignan	3	6.31 *s*	6.36 *s*	6.39 *s*
	6	6.99 *s*	6.95 *s*	6.95 *s*
	7	4.86 *d* (11.2)	4.92 *d* (14.0 Hz)	4.86 *d* (11.2)
	8	2.26 *m*	2.25 *m*	2.25*m*
	9	4.02 *m*<br>3.63 *dd* (4.8. 12.0)	3.67 *m*<br>3.96 *m*	4.00 *m*<br>3.62 *dd* (2.4. 11.2)
	10	5.82 *s*	5.87 *s*	5.90 *s*
	12. 13	1.42 *s*; 1.55 *s*	1.34 *s*; 1.51 *s*	1.44 *s*; 1.55 *s*
	2 ’6’	6.20 *s*	6.27 *s*	6.24 *s*
	7’	4.31 *bs*	4.37 *bs*	4.40 *bs*
	8’	2.71 *dd* (2.0. 4.8)	2.76 *m*	2.82 *dd* (2.0. 4.4)
	10’ 12’	3.76 *s*	3.74 *s*	3.75 *s*
	11’	3.71 *s*	3.70 *s*	3.72 *s*
**linker**		4.04 *m*<br>3.38 *m*<br>2.10 *m*	4.02 *m*<br>3.99 *m*<br>1.36-1.89 *m*	4.36 *m*<br>5.09 *d* (6.8)<br>*5.19 d* (6.8)<br>7.10 *d* (8.0)<br>7.30 *d* (8.0)

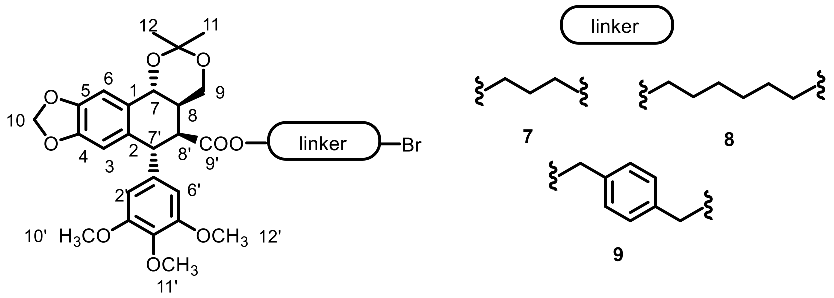

Table S6: ^{13}C NMR for compound **7-9**, δ in ppm.

	C	7	8	9
**Cyclolignan**	1	116.5	116.7	116.5
	2	130.6	130.8	130.8
	3	106.2	106.1	105.9
	4	147.4	147.1	147.3
	5	150.3	150.0	150.2
	6	109.3	109.5	109.6
	7	68.0	67.9	68.1
	8	29.0	28.9	28.7
	9	63.7	63.9	63.9
	10	101.0	100.9	100.7
	11	99.6	99.7	99.7
	12. 13	19.5. 29.7	19.5. 29.9	19.5. 29.8
	1’	140.8	140.6	140.6
	2’6’	104.8	104.7	104.6
	3’5’	153.3	153.1	153.2
	4’	141.8	141.9	141.7
	7’	47.0	46.8	47.1
	8’	35.2	34.9	34.8
	9’	172.7	172.9	172.9
	10’ 12’	59.7	59.7	59.7
	11’	60.9	60.8	61.0
**linker**		39.2. 63.1. 65.0	28.3. 28.8. 35.3.<br>38.9. 63.1. 64.9	63.4. 65.7. 125.6. 125.8.<br>128.2. 128.7.136.4. 136.7

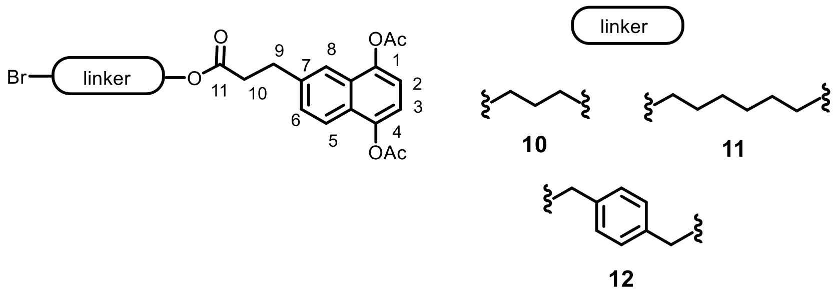

Table S7: ^{1}H NMR for compound **10-12**, δ in ppm (*J* in Hz)

**H**	**10**	**11**	**12**
**2**	7.21 *d* (8.0)	7.21 *d* (8.0)	7.22 *d* (8.0)
**3**	7.17 *d* (8.0)	7.17 *d* (8.0)	7.20 *d* (8.0)
**C_1-CO-O$\underline{CH_3}$**	2.39 *s*	2.39 *s*	2.46 *s*
**C_4-CO-O$\underline{CH_3}$**	2.37 *s*	2.37 *s*	2.46 *s*
**5**	7.71 *d* (8.8)	7.71 *d* (8.8)	7.80 *d* (8.8)
**7**	7.32 *dd* (1.8; 8.8)	7.32 *dd* (1.8; 8.8)	7.38 *dd* (1.8; 8.8)
**8**	7.66 *d* (1.8)	7.66 *d* (1.8)	7.66 *d* (1.8)
**9**	3.03 *t* (8)	3.03 *t* (8)	3.15 *t* (7.6)
**10**	2.60 *t* (8)	2.60 *t* (8)	2.78 *t* (7.6)
**C_{11}OOR**	4.04 *t* (7.2)<br>1.96 *m*<br>3.22 *t* (6.4)	3.97 *t* (6.8)<br>1.71 *m*<br>1.27 *m*<br>1.19 *m*<br>1.48 *m*<br>3.28 *t* (7.2)	5.10 *s*<br>7.33 *d* (8.4)<br>7.26 *d* (8.4)<br>4.47 *s*

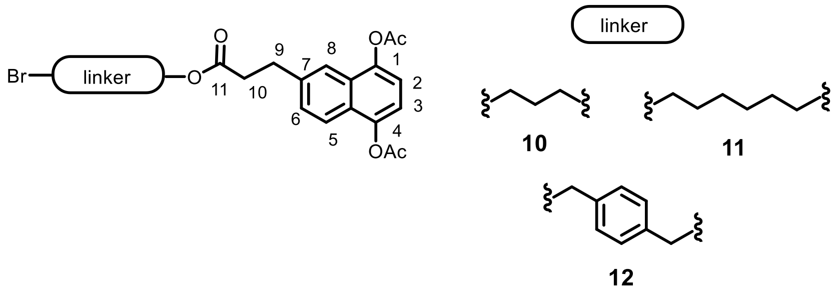

Table S8: ^{13}C NMR for compound **10-12**, δ in ppm.

C	10	11	12
**1**	144.7	147.2	147.5
**2**	118.1	117.1	117.9
**3**	117.2	117.4	117.1
**4**	144.5	147.0	147.2
**4a**	126.7	126.4	127.1
**5**	122.9	122.2	122.3
**6**	129.0	128.3	129.2
**7**	142.5	140.9	140.5
**8**	120.4	120.2	120.3
**8a**	128.3	128.0	127.9
**9**	34.1	35.1	35.6
**10**	33.1	31.5	31.2
**11**	177.8	177.8	177.3
**-O<u>C</u>OCH$_3$**	169.9	169.6	169.4
**-OCO<u>CH$_3$</u>**	21.5	21.2	20.0
**C$_{11}$OO<u>R</u>**	29.1<br>35.5<br>64.5	27.4<br>27.7<br>38.4<br>33.8<br>64.8	31.1<br>127.0<br>128.7<br>129.4<br>133.3<br>136.6

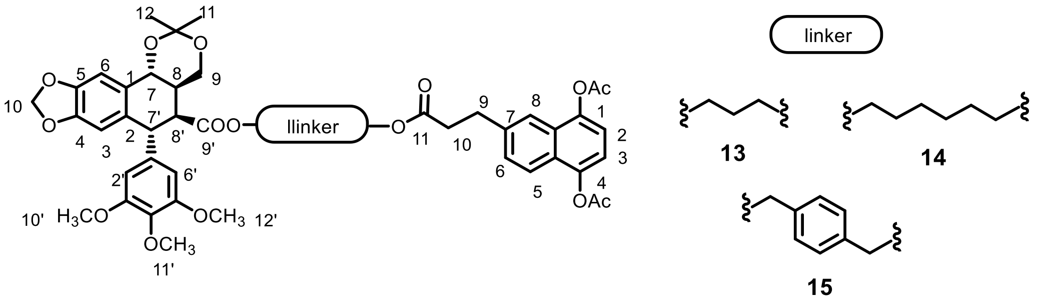

Table S9: ^{1}H NMR for compound **13-15**, δin ppm (*J* in Hz)

H		**13**	**14**	**15**
**MHQ**	2	7.21 *d* (8.0)	7.21 *d* (8.0)	7.22 *d* (8.0)
	3	7.17 *d* (8.0)	7.17 *d* (8.0)	7.18 *d* (8.0)
	5	4.90 *s*	7.78 *d* (8.8)	7.78 *d* (8.0)
	7	1.19 *s*	7.40 *dd* (2.0; 8.8)	7.39 *dd* (1.8; 8.0)
	8	0.43 *s*	7.64 *d* (2.0)	7.66 *d* (1.8)
	9	7.12 *d* (8.0)	3.11 *t* (7.6)	3.14 *t* (7.6)
	10	7.14 *d* (8.0)	2.66 *t* (7.6)	2.82 *t* (7.6)
	-OAc	2.44 *s*; 2.45 *s*	2.44 *s*. 2.45 *s*	2.45 *s*
**linker**		4.02 *m*<br>1.80 *m*<br>1.25 *m*	4.02 *m*<br>3.99 *m*<br>1.25-1.80 *m*	5.19 *d* (10.4)<br>5.09 *d* (10.4)<br>7.24 *d* (7.6)<br>7.10 *d* (7.6)
**Cyclolignan**	3	6.38 *s*	6.38 *s*	6.39 *s*
	6	6.97 *s*	6.97 *s*	6.95 *s*
	7	4.90 *d* (10.8)	4.90 *d* (10.8)	4.75 *d* (10.8)
	8	2.17 *m*	2.17 *m*	2.25 *m*
	9	4.04 *m*<br>3.68 *dd*<br>(4.4; 11.2)	4.04 *m*<br>3.68 *dd*<br>(4.4; 11.2)	3.96 *t* (11.6)<br>3.63 *dd*<br>(3.6; 11.6)
	10	5.89 *s*	5.89 *s*	5.90 *s*
	12. 13	1.49 *s;* 1.55 *s*	1.49 *s;* 1.55 *s*	1.34 *s;* 1.51 *s*
	2 ’6’	6.25 *s*	6.25 *s*	6.24 *s*
	7’	4.72 *d* (10.2)	4.72 *d* (10.2)	4.74 *d* (10.8)
	8’	2.78 *m*	2.78 *m*	2.25 *m*
	10’ 12’	3.78 *s*	3.78 *s*	3.74 *s*
	11’	3.83 *s*	3.83 *s*	3.79 *s*

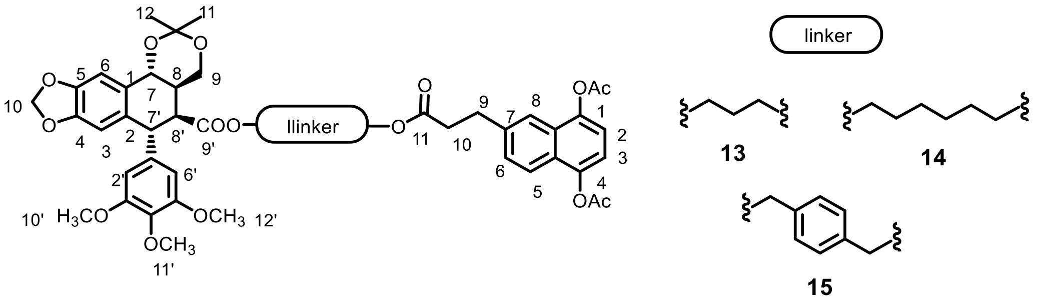

Table S10: ^{13}C NMR for compound **13-15**, δ in ppm.

	C	13	14	15
**MHQ**	1	147.0	147.2	147.5
	2	117.9	118.1	117.9
	3	117.2	117.4	117.1
	4	147.0	147.0	147.2
	4a	126.3	126.4	127.1
	$C_1CO\text{-}OCH_3$	169.3; 21.0	169.6; 21.3	169.4; 20.0
	$C_4CO\text{-}OCH_3$	169.3; 21.0	169.6; 21.2	169.6; 20.0
	5	122.0	122.2	122.3
	6	128.4	128.3	129.2
	7	140.5	140.9	140.5
	8	120.3	120.2	120.3
	8a	128.0	128.0	127.9
	9	35.6	35.1	35.6
	10	31.6	31.5	31.2
	11	122.0	122.2	122.3
**linker**		35.2; 65.8; 66.3	65.0; 64.6; 35.1 28.7; 25.7; 25.6	136.2; 135.4; 128.7; 128.7; 67.2; 66.9
**Cyclolignan**	1	126.3	126.6	128.0
	2	130.8	131.1	130.8
	3	105.1	105.0	104.7
	4	147.1	147.2	147.0
	5	150.0	147.2	146.9
	6	109.5	109.7	109.5
	7	67.9	68.1	67.8
	8	29.0	35.8	34.9
	9	63.7	63.3	62.9
	10	101.0	99.9	101.0
	11	99.6	98.7	99.6
	12. 13	30.0; 19.6	30.1; 19.8	29.9; 19.3
	1’	137.3	138.2	140.5
	2’6’	106.4	106.4	106.1
	3’5’	152.9	153.3	153.1
	4’	137.9	137.7	136.8
	7’	46.8	47.0	46.6
	8’	68.0	68.1	67.8
	9’	172.1	173.1	172.4
	10’ 12’	56.2	56.4	60.8
	11’	60.8	61.1	62.9

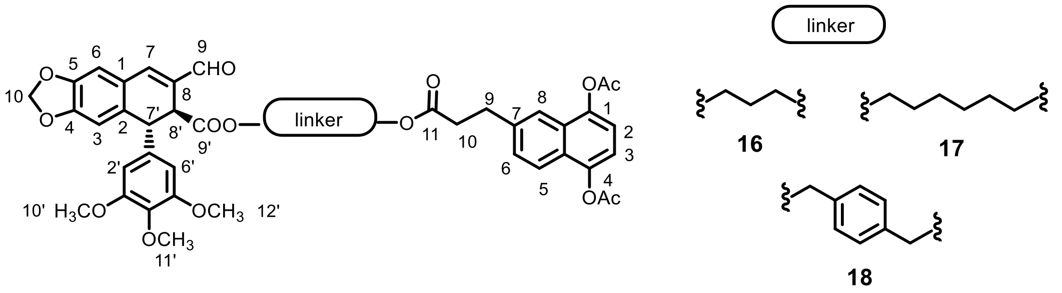

Table S11: ^{1}H NMR for compound **16-18**, δ in ppm (*J* in Hz)

	H	16	17	18
**MHQ**	2	7.21 *d* (8.0)	7.20 d (8.0)	7.22 *d* (8.0)
	3	7.18 *d* (8.0)	7.17 d (8.0)	7.18 *d* (8.0)
	5	7.78 *d* (8.0)	7.78 *d* (9.0)	7.78 *d* (8.0)
	7	7.39 *dd* (1.8; 8.0)	7.39 *dd* (0.8; 9.0)	7.39 *dd* (1.8; 8.0)
	8	7.64 *d* (1.8)	7.66 *d* (0.8)	7.66 *d* (1.8)
	9	3.08 *t* (8.0)	3.01 *t* (7.6)	3.13 *t* (7.6)
	10	2.66 *t* (8.0)	2.67 *t* (7.6)	2.74 *t* (7.6)
	-OAc	2.43 *s*; 2.45 *s*	2.43 *s*. 2.45 *s*	2.44 *s*. 2.45 *s*
**linker**		4.06 t (6.0)<br>1.88 m<br>3.98 t (4.0)	4.02 *t* (6.8)<br>1.54 *m*<br>1.46 *m*<br>1.23 *m*<br>1.20 *m*<br>3.97 *m*	5.08 d (10.8)<br>5.06 d (10.8)<br>7.24 d (7.6)<br>7.10 d (7.6)<br>5.09 m
**Cyclolignan**	3	6.47 *s*	6.50 *s*	6.61 *s*
	6	6.87 *s*	6.87 *s*	6.88 *s*
	7	7.33 *s*	7.41 *s*	7.38 *s*
	9	9.59 *s*	9.59 s	9.59 *s*
	10	5.98 *s*	5.98 *s*	6.00 *s*
	2 . 6’	6.21 *s*	6.20 *s*	6.20 *s*
	7’	4.59 *d* (4.0)	4.59 *d* (3.2)	4.58 *d* (4)
	8’	2.82 *d* (3.6)	3.97 *m*	4.04 *d* (4)
	10’.12’	3.73 *s*	3.73 *s*	3.76 *s*
	11’	3.75 *s*	3.76 *s*	3.81 *s*

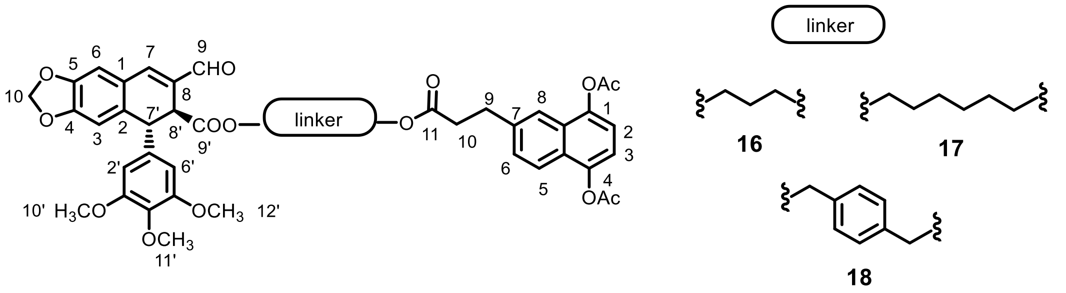

Table S12: ^{13}C NMR for compound **16-18**, δ in ppm.

	C	16	17	18
**MHQ**	1	144.3	145.5	144.3
	2	117.9	119.4	118.1
	3	117.1	118.6	117.4
	4	144.0	145.5	144.0
	4a	126.4	129.7	122.2
	$C_1CO-OCH_3$	169.4; 21.0	170.9; 22.6	169.5; 21.3
	$C_4CO-OCH_3$	169.4; 21.0	170.9; 22.6	169.6; 21.4
	5	122.0	123.5	122.2
	6	128.0	129.3	128.0
	7	147.4	141.0	137.1
	8	120.1	121.7	120.2
	8a	127.7	129.7	182.1
	9	31.1	37.2	35.6
	10	27.7	32.8	31.1
	11	171.6	173.2	171.6
**linker**		35.5<br>60.7<br>61.6	25.2 28.4 38.0<br>39.0 63.9 65.1	133.7<br>128.3<br>127.8<br>133.1<br>66.0<br>66.5
**Cyclolignan**	1	125.0	126.7	125.3
	2	133.1	135.3	133.8
	3	110.0	111.6	117.1
	4	150.5	151.9	150.4
	5	108.9	151.9	150.1
	6	147.4	110.4	110.0
	7	133.7	146.9	145.6
	8	191.2	134.8	133.1
	9	101.8	192.8	191.2
	10	125.0	103.3	101.8
	1’	137.0	35.6	135.7
	2’. 6’	104.8	107.0	104.8
	3’. 5’	153.2	154.7	153.2
	4’	139.4	138.7	136.9
	7’	46.4	47.8	46.4
	8’	44.7	46.1	44.4
	9’	172.4	174.2	172.4
	10’. 12’	60.7	57.7	56.1
	11’	56.1	62.3	60.7

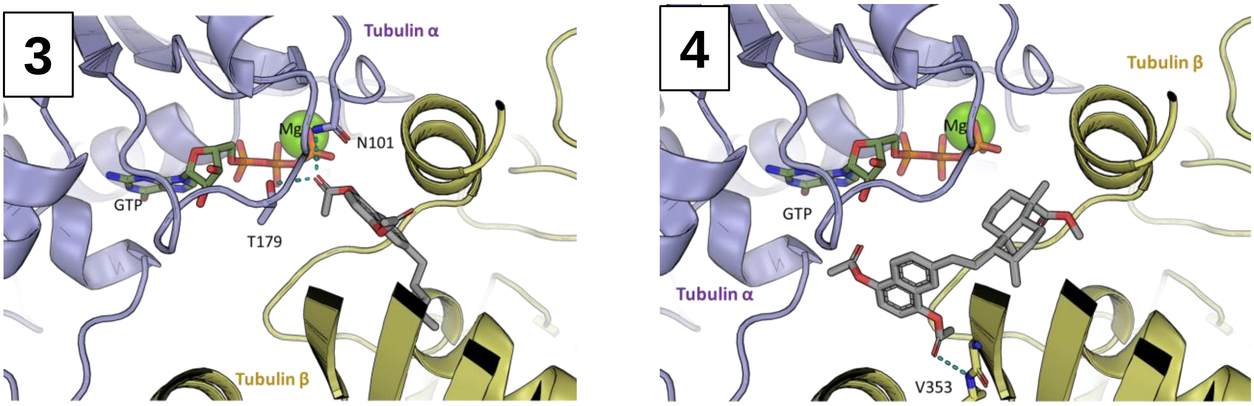

Figure S1: Docking complexes of precursors **3** and **4** in the colchicine site of tubulin. GTP, the inhibitors, and those residues that interact with the inhibitors via hydrogen bonds are shown atomistically. The two protomers tubulin and are shown in yellow and purple, respectively

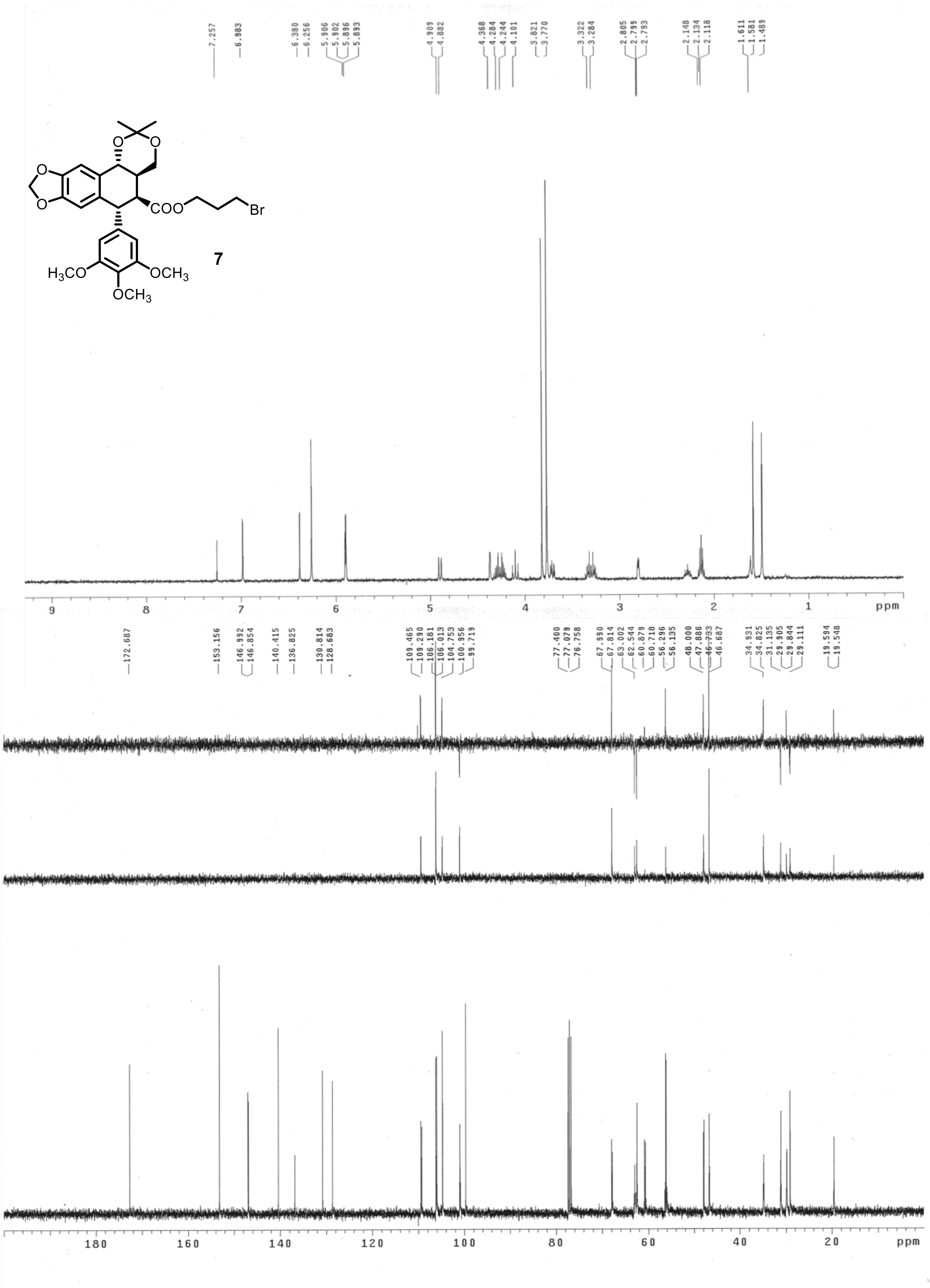

Figure S2: ^{1}H and ^{13}C NMR spectra for compound **7**

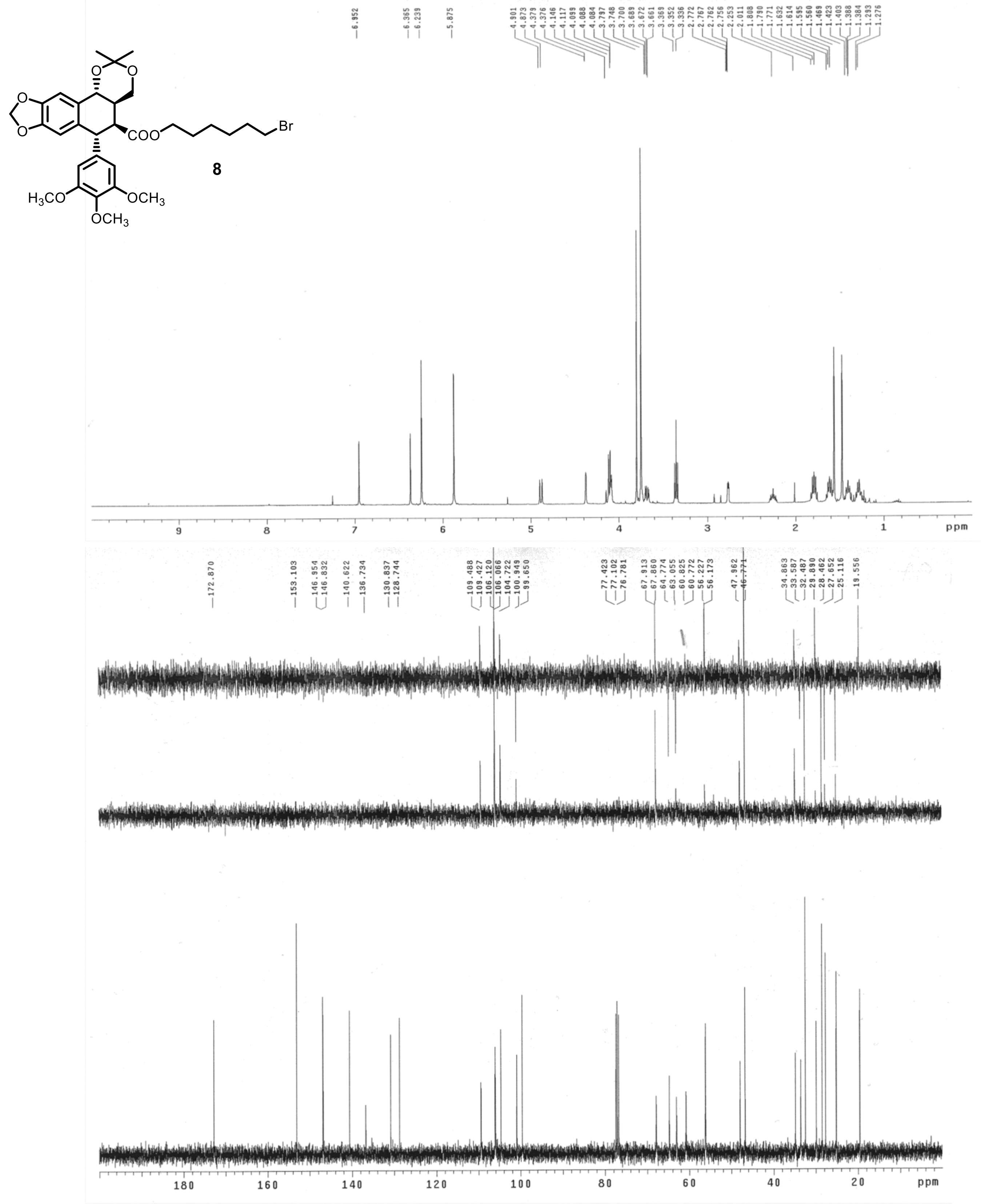

Figure S3: ^{1}H and ^{13}C NMR spectra for compound **8**

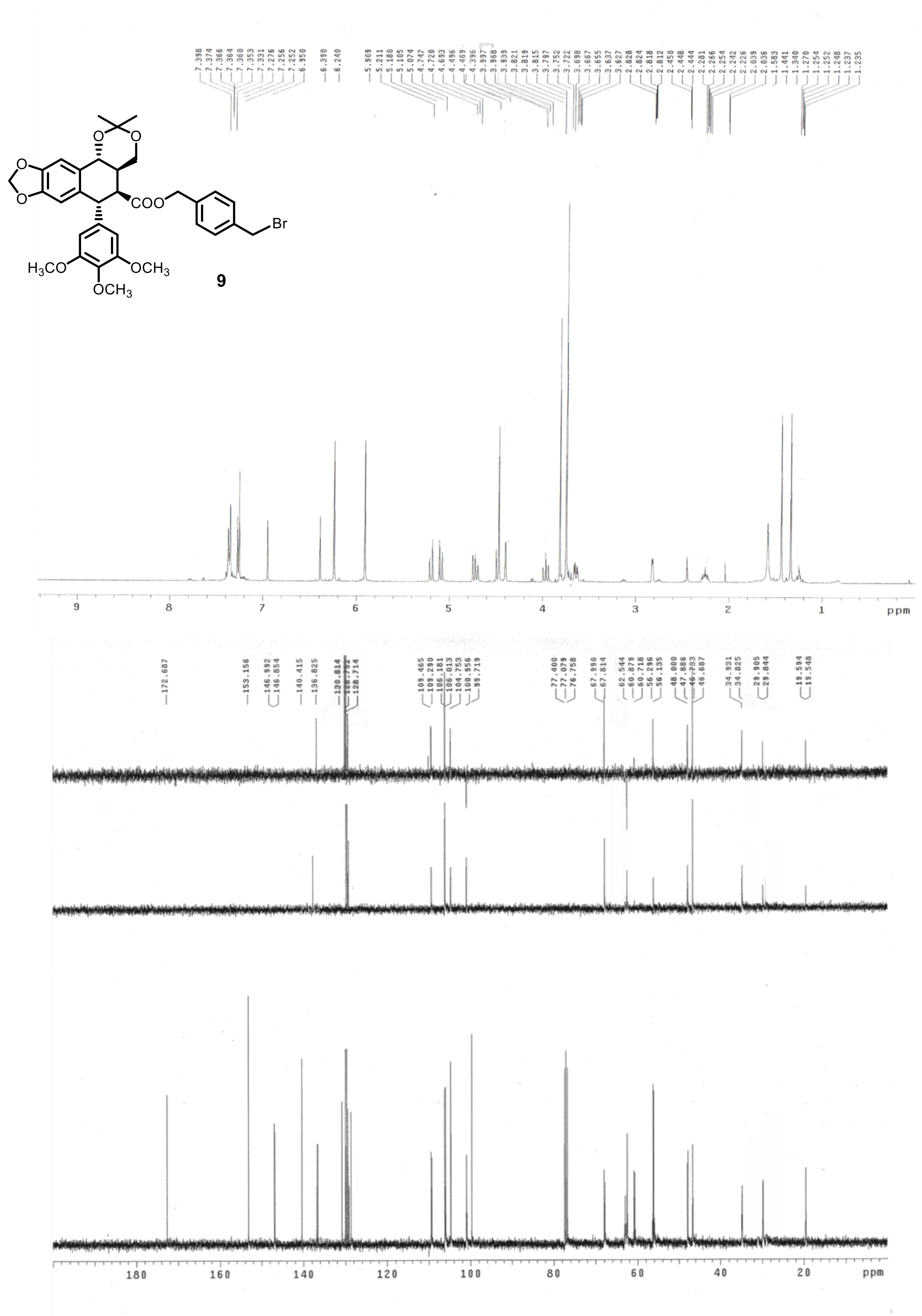

Figure S4: ^{1}H and ^{13}C NMR spectra for compound **9**

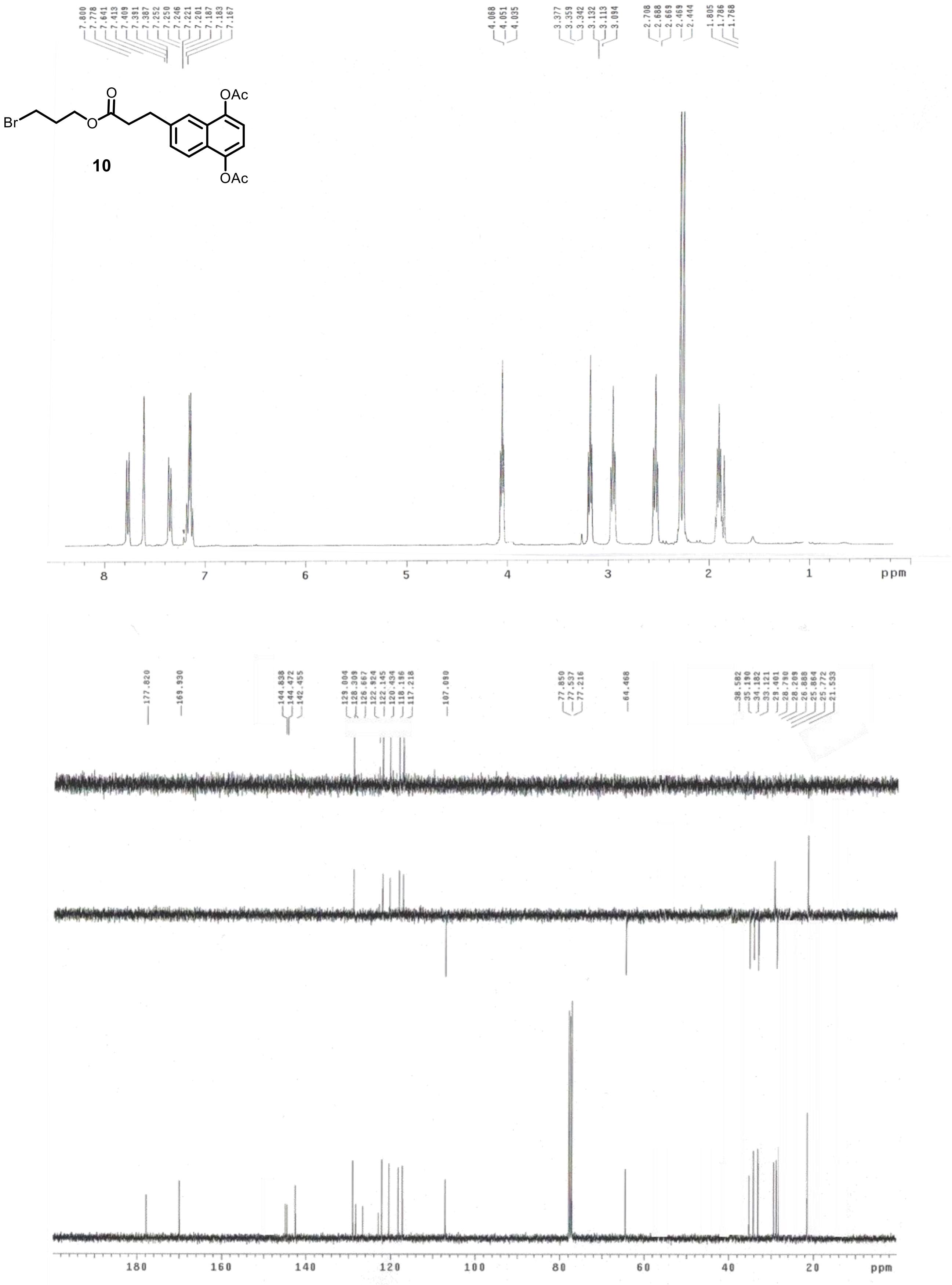

Figure S5: ^{1}H and ^{13}C NMR spectra for compound **10**

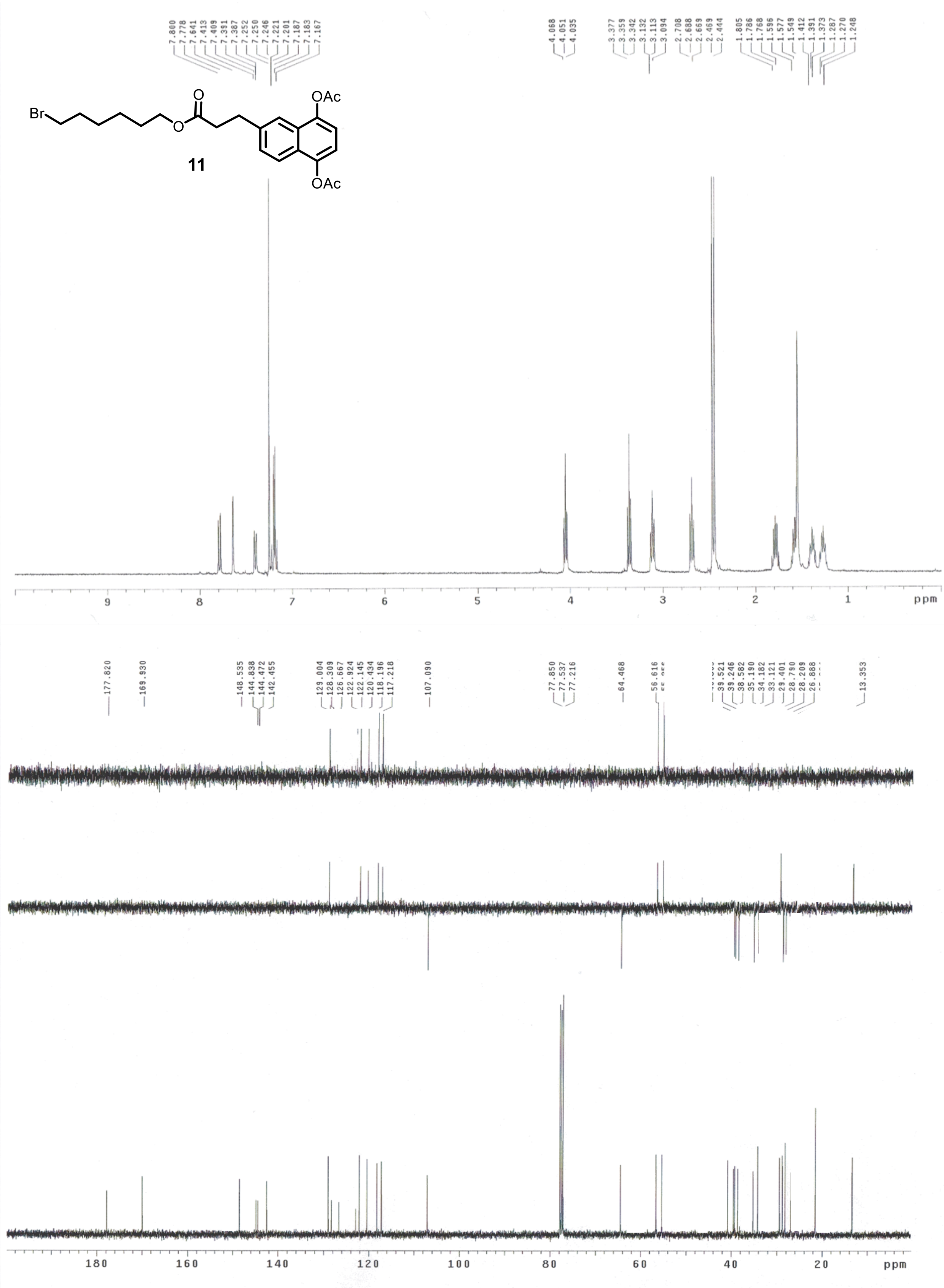

Figure S6: ^{1}H and ^{13}C NMR spectra for compound **11**

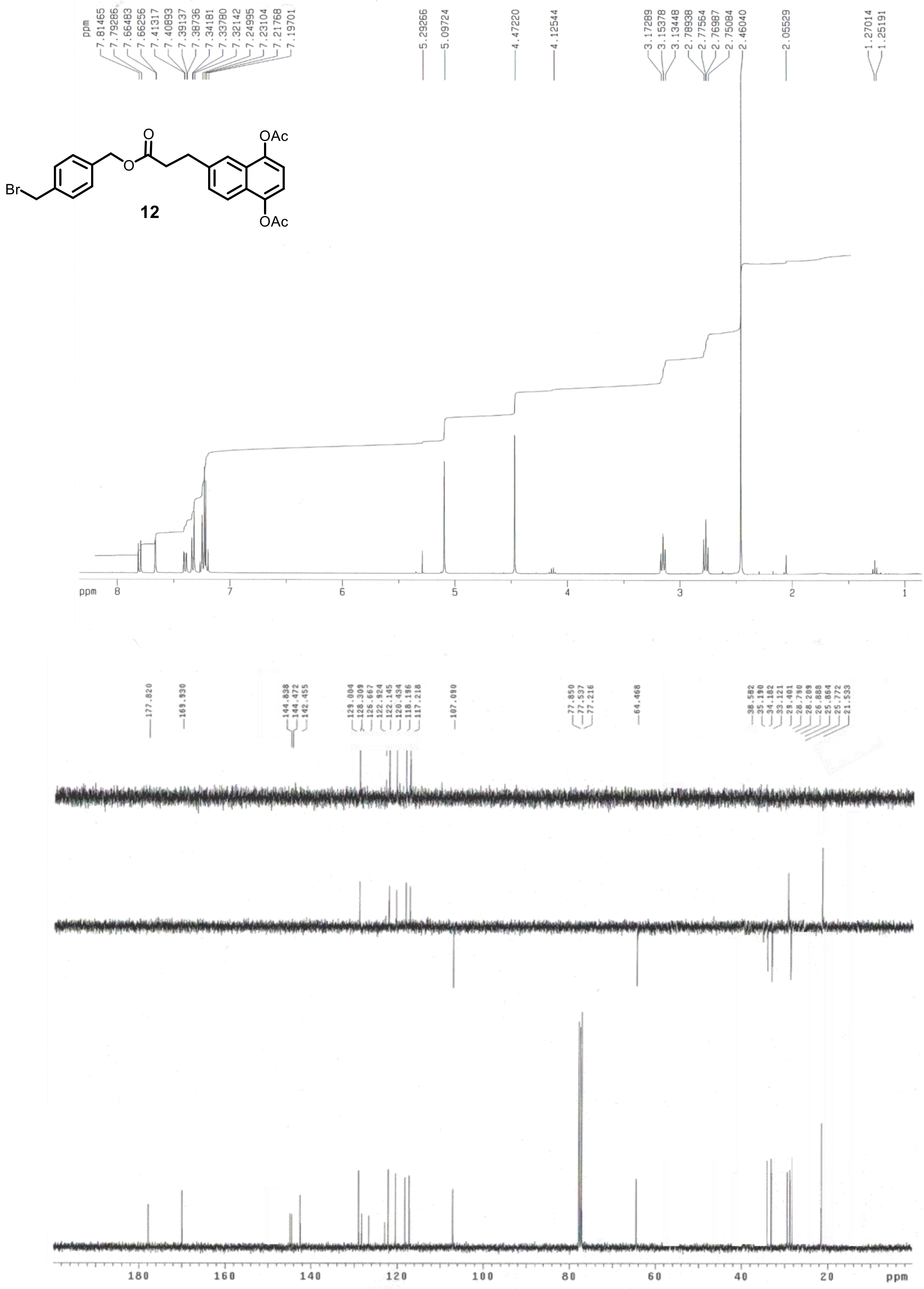

Figure S7: ^{1}H and ^{13}C NMR spectra for compound **12**

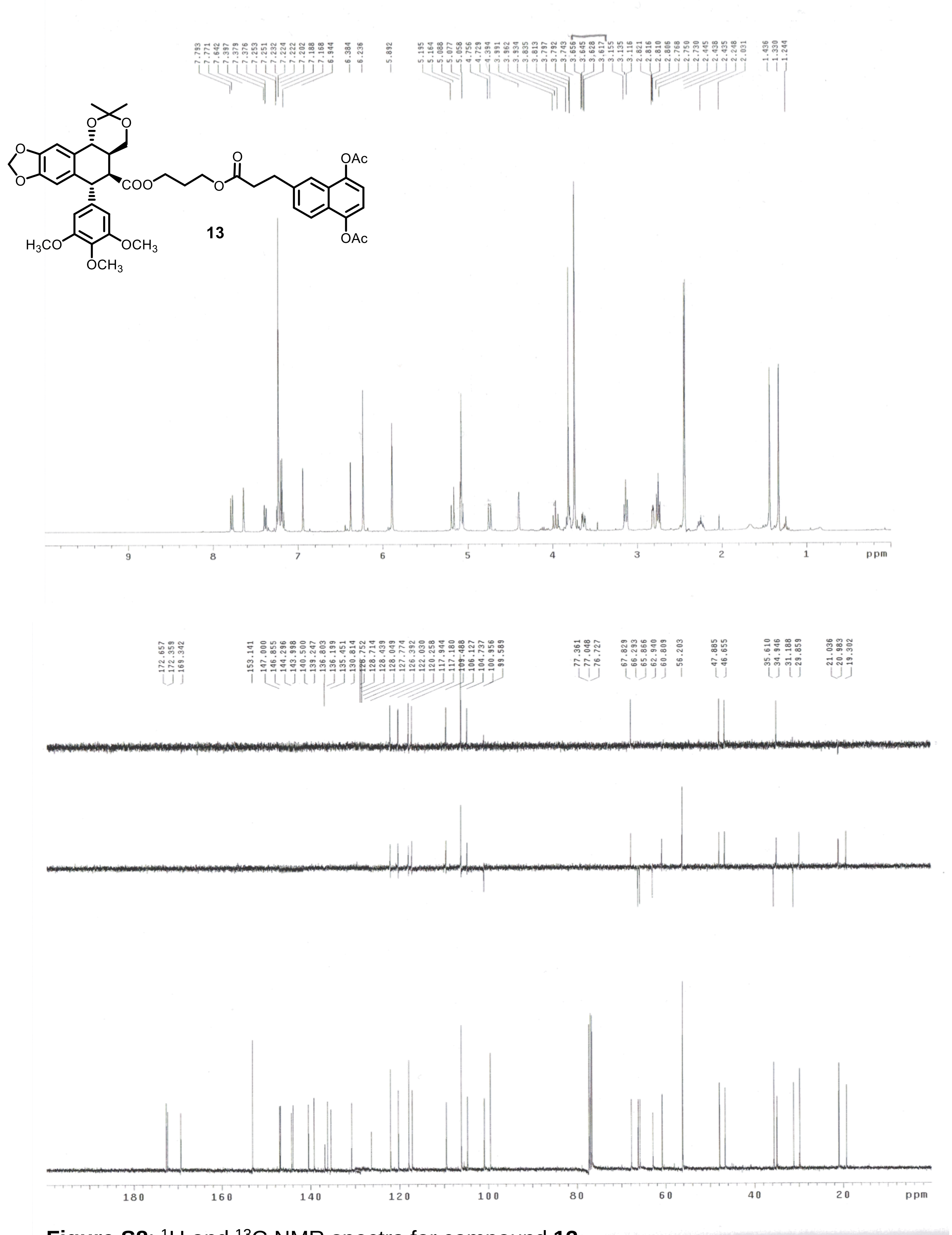

13

Figure S8: ^{1}H and ^{13}C NMR spectra for compound **13**

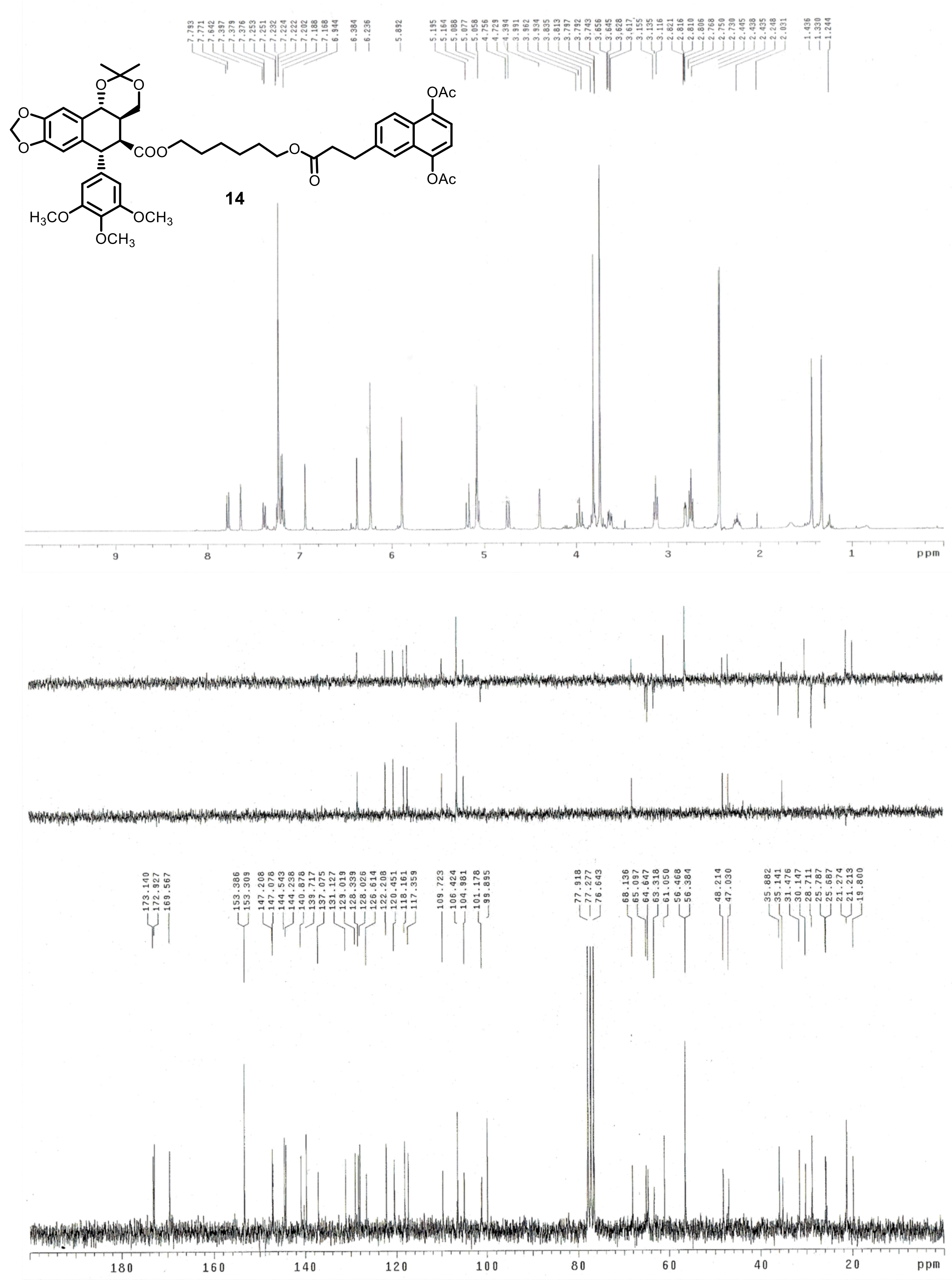

Figure S9: ^{1}H and ^{13}C NMR spectra for compound **14**

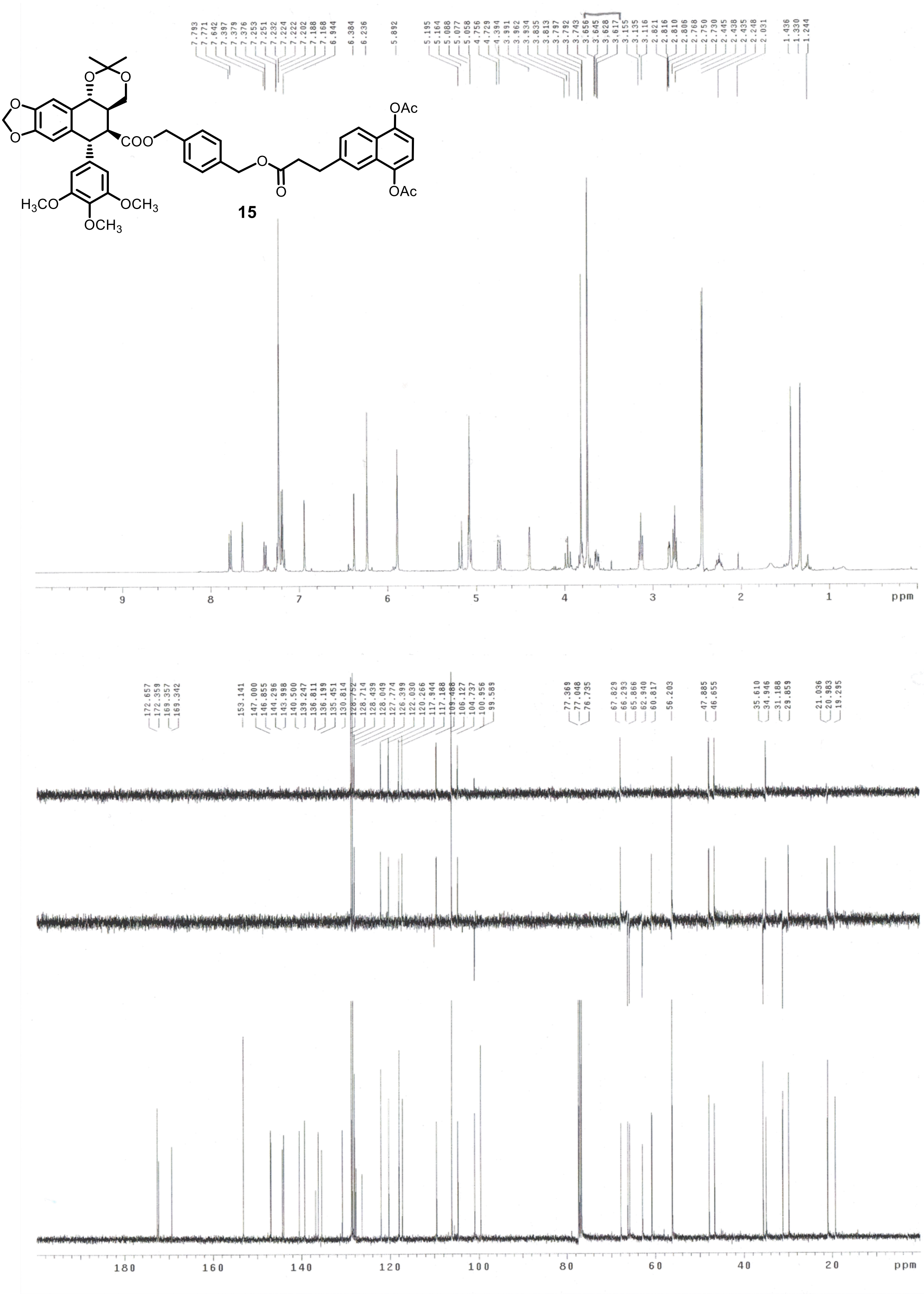

Figure S10: ^{1}H and ^{13}C NMR spectra for compound **15**

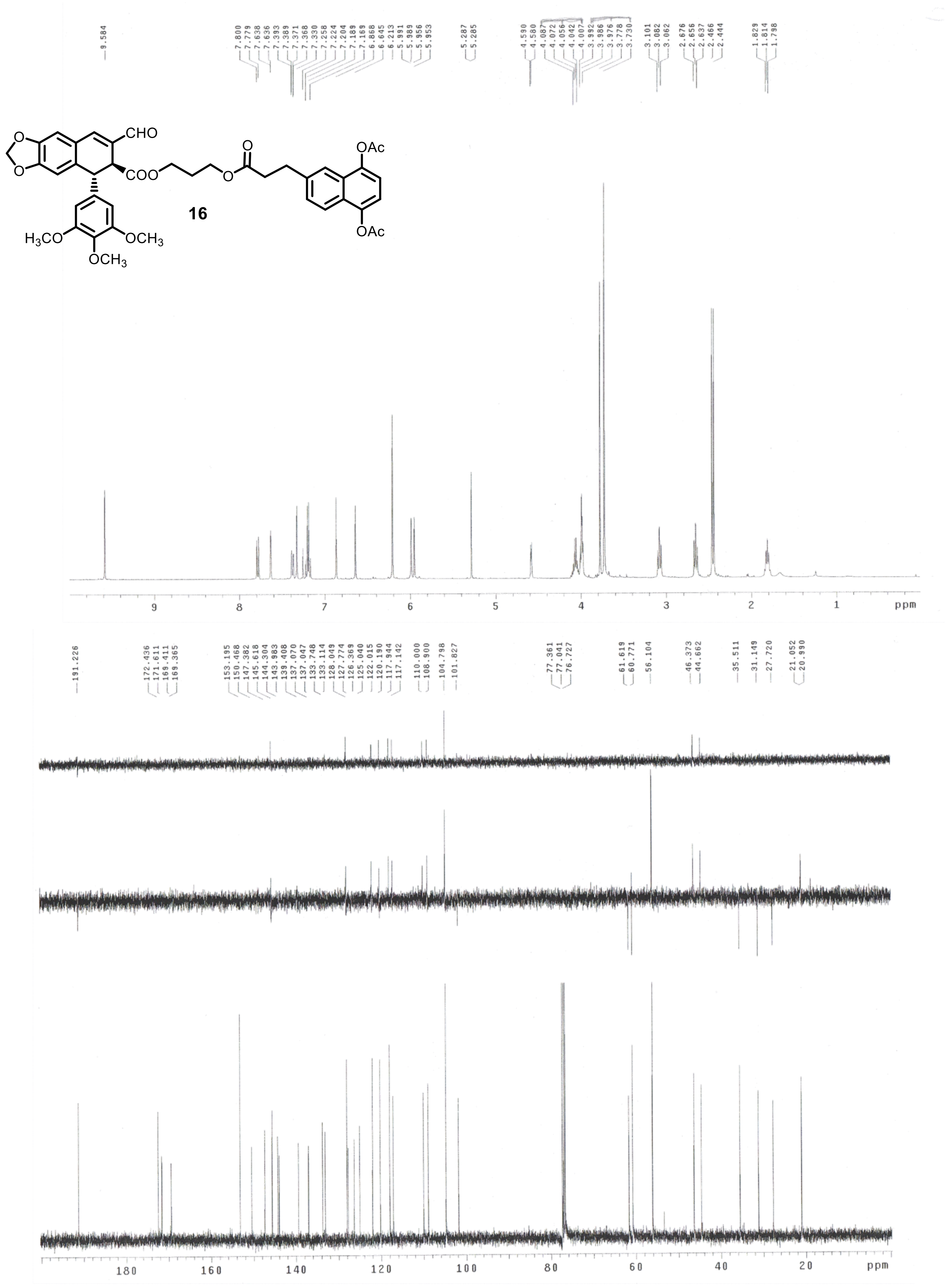

Figure S11: ^{1}H and ^{13}C NMR spectra for compound **16**

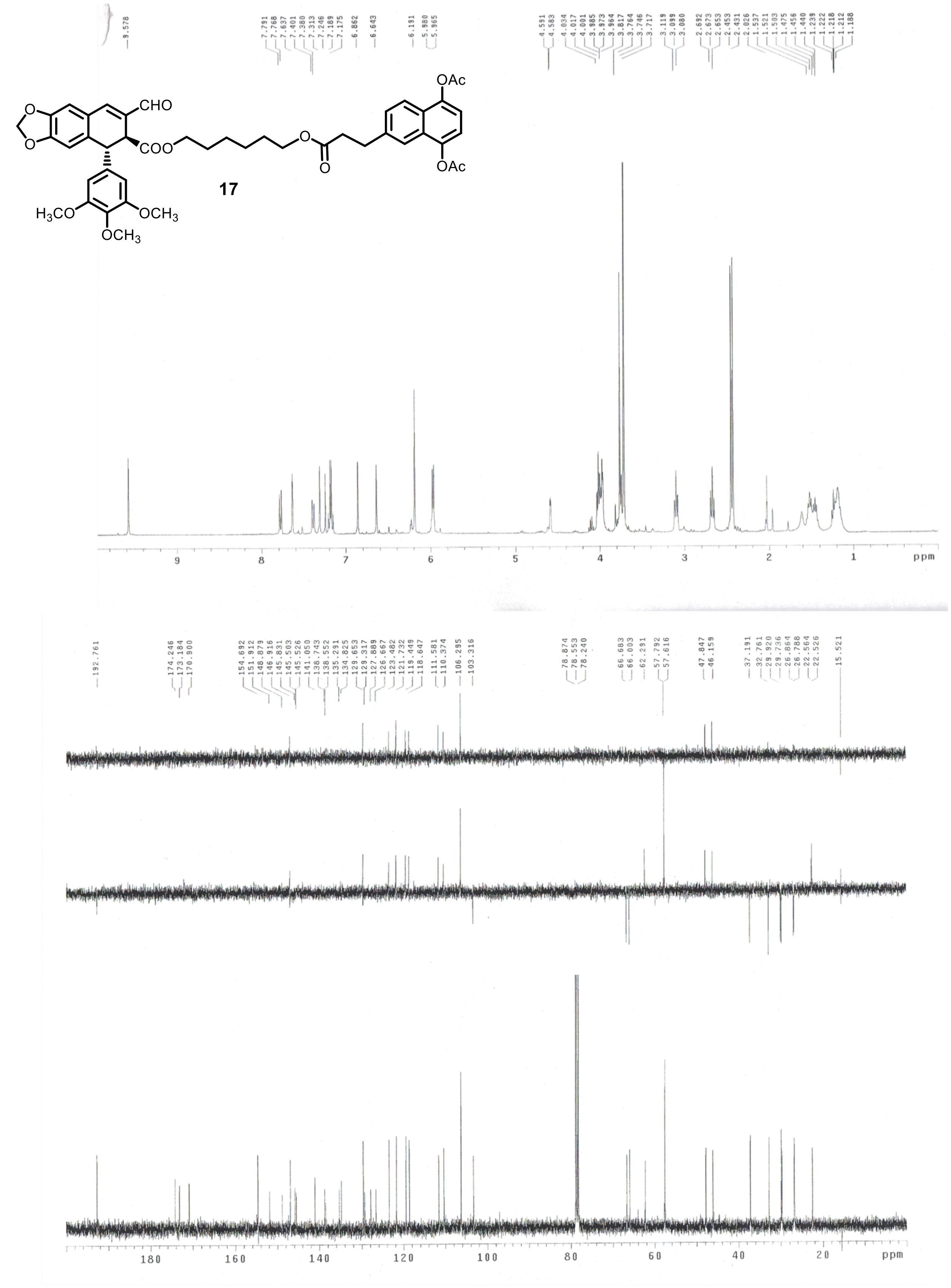

Figure S12: ^{1}H and ^{13}C NMR spectra for compound **17**

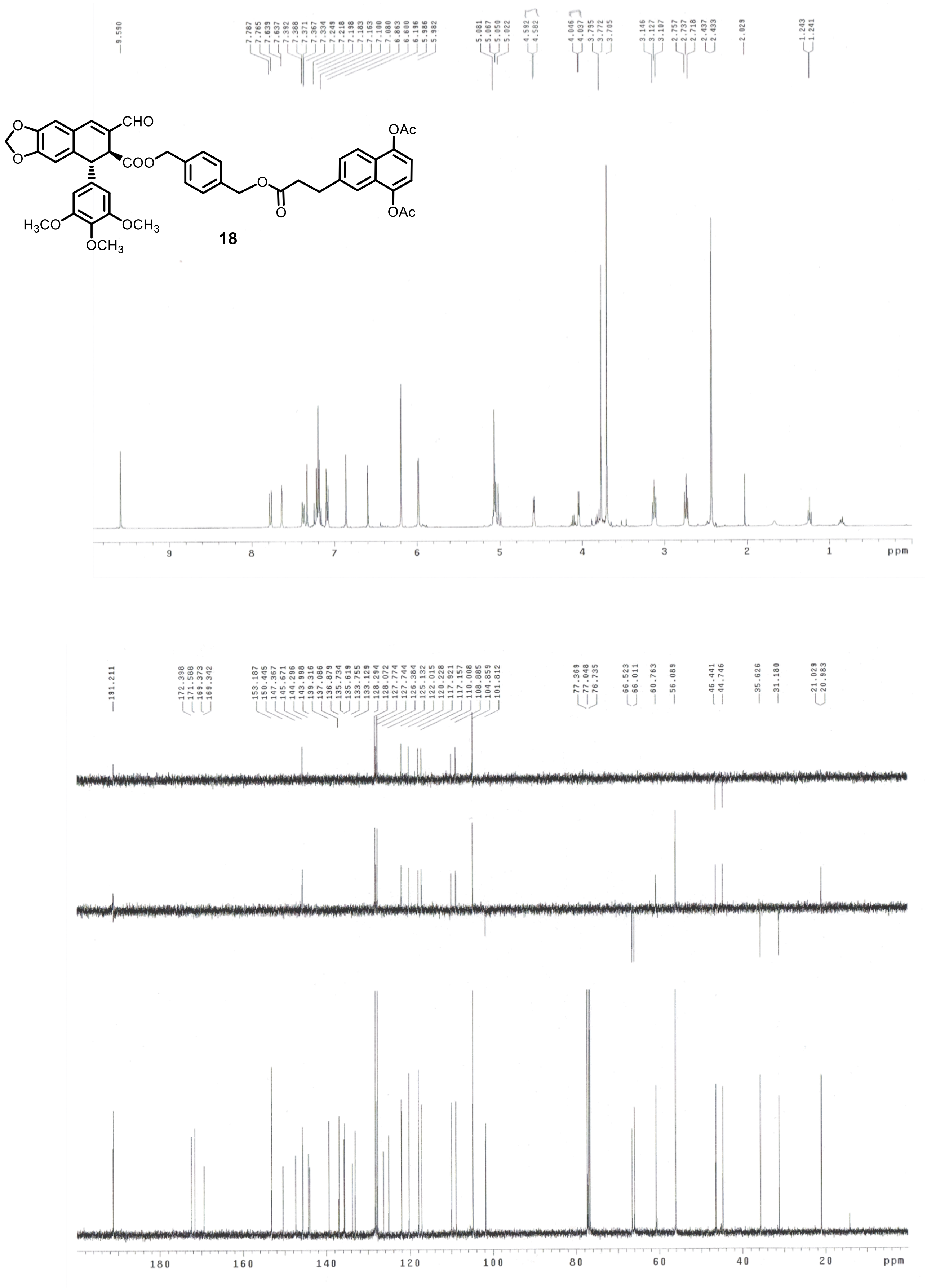

Figure S13: ^{1}H and ^{13}C NMR spectra for compound **18**
